# Drug screening platform using human induced pluripotent stem cell‐derived atrial cardiomyocytes and optical mapping

**DOI:** 10.1002/sctm.19-0440

**Published:** 2020-09-14

**Authors:** Marvin G. Gunawan, Sarabjit S. Sangha, Sanam Shafaattalab, Eric Lin, Danielle A. Heims‐Waldron, Vassilios J. Bezzerides, Zachary Laksman, Glen F. Tibbits

**Affiliations:** ^1^ Molecular Cardiac Physiology Group, Departments of Biomedical Physiology and Kinesiology and Molecular Biology and Biochemistry Simon Fraser University Burnaby British Columbia Canada; ^2^ Tibbits Research Team British Columbia Children's Hospital Research Institute Vancouver British Columbia Canada; ^3^ Division of Cardiology, Faculty of Medicine University of British Columbia Vancouver British Columbia Canada; ^4^ Department of Cardiology Boston Children's Hospital Boston Massachusetts USA; ^5^ Centre for Heart and Lung Innovation, St. Paul's Hospital Vancouver British Columbia Canada

**Keywords:** atrial differentiation, atrial fibrillation, cardiomyocyte subtype, drug screening, human induced pluripotent stem cells

## Abstract

Current drug development efforts for the treatment of atrial fibrillation are hampered by the fact that many preclinical models have been unsuccessful in reproducing human cardiac physiology and its response to medications. In this study, we demonstrated an approach using human induced pluripotent stem cell‐derived atrial and ventricular cardiomyocytes (hiPSC‐aCMs and hiPSC‐vCMs, respectively) coupled with a sophisticated optical mapping system for drug screening of atrial‐selective compounds in vitro. We optimized differentiation of hiPSC‐aCMs by modulating the WNT and retinoid signaling pathways. Characterization of the transcriptome and proteome revealed that retinoic acid pushes the differentiation process into the atrial lineage and generated hiPSC‐aCMs. Functional characterization using optical mapping showed that hiPSC‐aCMs have shorter action potential durations and faster Ca^2+^ handling dynamics compared with hiPSC‐vCMs. Furthermore, pharmacological investigation of hiPSC‐aCMs captured atrial‐selective effects by displaying greater sensitivity to atrial‐selective compounds 4‐aminopyridine, AVE0118, UCL1684, and vernakalant when compared with hiPSC‐vCMs. These results established that a model system incorporating hiPSC‐aCMs combined with optical mapping is well‐suited for preclinical drug screening of novel and targeted atrial selective compounds.

## Graphical abstract

Chamber‐specific differentiation of hiPSC‐derived cardiomyocytes allowed for the development of an atrial cardiomyocyte‐based drug screening platform. In‐depth characterization of hiPSC‐derived atrial and ventricular cardiomyocytes revealed chamber‐specific phenotypes in molecular signatures and functional profiles. Drug screening with high‐content optical mapping system captured atrial‐selective pharmacology which demonstrated the utility of hiPSC‐derived atrial cardiomyocytes as an in vitro drug screening model.

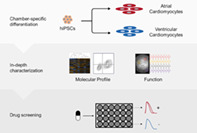

**Significance statement**

Current in vitro drug screening systems for treatment of atrial fibrillation are confounded by cell type heterogeneity, specificity, and translatability to human physiology. In this study, a drug screening platform was developed using human induced pluripotent stem cell‐derived atrial cardiomyocytes (hiPSC‐aCMs) and a multiwell optical mapping system. The high‐content optical mapping system reports on membrane voltage and Ca^2+^ transients, which serve as critical biomarkers of cardiac function in vitro. The hiPSC‐aCMs generated by this protocol possess atrial‐specific molecular profiles, functional signatures, and pharmacological response. These findings demonstrate that the platform can be readily applied as a relevant preclinical model for drug screening for atrial fibrillation therapies.

## 1 INTRODUCTION

The advent of human induced pluripotent stem cell‐derived cardiomyocytes (hiPSC‐CMs) has revolutionized the field of cardiac research. It has enabled the study of cardiac diseases in a patient‐specific and human‐relevant in vitro model system which provides a unique opportunity for clinical translation.^1^ Furthermore, the ability to differentiate chamber‐specific cardiomyocytes allows for a more precise study of cardiac disease physiology and pharmacology.

The cardiomyocytes of the lower (ventricles) and upper (atria) chambers have distinct characteristics that arise from differential developmental pathways. Previous work in vivo has shown that the expression patterns of retinoic acid and retinaldehyde dehydrogenase 2 (RALDH2) are important determinants of the atrial fate.^2^, ^3^, ^4^, ^5^ These results were later recapitulated in a pivotal study by Lee and Protze et al^6^ who determined that atrial cardiomyocytes (aCMs) differentiated from human embryonic stem cells (hESCs) originate from a unique mesoderm characterized by robust RALDH2 expression. This study established an atrial differentiation protocol that included the addition of retinoic acid. Retinoic acid has also been utilized to selectively differentiate hESCs and hiPSCs into aCMs in other studies.^6^, ^7^, ^8^, ^9^, ^10^

The distinct properties of the atrial and ventricular cardiomyocytes are determined by the differential expression of unique sets of ion channels and other proteins that optimize their specific function. Drugs that target atrial ion channels selectively can therefore produce differences in pharmacological function in the two chambers. This atrial‐selective pharmacology is of utmost interest in the study and treatment of atrial‐specific diseases such atrial fibrillation (AF), which is the most common heart rhythm disorder. Investigating atrial‐selective pharmacology can assist and guide novel cardiac drug development as well as improving both safety and efficacy by avoiding potential toxic electrophysiologic effects on the ventricular chambers.

The differential pharmacology of stem cell‐derived aCMs was studied previously by Laksman et al^7^ who showed that flecainide can rescue the AF phenotype in a dish. Other studies have also studied the selective pharmacological effects of agents on hiPSC‐derived aCMs but have largely focused on proof‐of‐concepts using limited number of test compounds and standard measurement systems that are low in throughput.^9^, ^10^ With a focus on translation, a preclinical model platform that characterizes pharmacological activity must capture the main cardiac functional signatures that most closely mimic and predict human cardiac physiology and drug responses. As such, we established in this study an in vitro assay platform by combining hiPSC‐derived atrial cardiomyocytes (hiPSC‐aCMs) and high‐content optical mapping, a noninvasive all‐optical system that simultaneously measures membrane potential (V_m_) and Ca^2+^ transients at a high‐resolution in a monolayer tissue format.

We first demonstrate a selective hiPSC‐aCM differentiation protocol by modifying the well characterized GiWi protocol^11^ through the controlled introduction of retinoic acid. The recapitulation of the human atrial phenotype of the hiPSC‐aCMs was validated with assays that measure the expression of gene transcripts and proteins, as well as functional signatures. We then demonstrate the utility of our platform as an atrial‐selective drug screening tool by using existing clinical and experimental drugs. The model established in this study adds to our current understanding of the utility of stem cell‐derived cardiomyocytes in preclinical and translational research focused on screening new pharmacological agents.

## 2 MATERIALS AND METHODS

A detailed methods section is available in the Supplemental Information.

### 2.1 Maintenance and expansion of hiPSCs

hiPSCs (WiCell, IMR90‐1) were maintained and expanded in mTeSR1 medium and feeder‐free culture using 6‐well plates coated with Matrigel. Using Versene (EDTA), hiPSCs were passaged every 4 days or ~85% confluency at 1:15 ratio. Passaged hiPSCs were cultured with mTeSR1 supplemented with 10 μM Y27632 for the first 24 hours and the mTeSR1 was exchanged daily during cell culture maintenance.

### 2.2 Directed differentiation of hiPSCs into atrial and ventricular subtypes

hiPSC‐derived ventricular cardiomyocytes were differentiated by employing a modified GiWi protocol^11^ that we previously published.^12^ In brief, hiPSCs were seeded at a density of 87 500 cells/cm^2^. At day 0, differentiation was initiated using 12 μM CHIR99021. At day 3, the cells were incubated with 5 μM IWP‐4. At day 5, the media were refreshed with RPMI‐1640 supplemented with B27 minus insulin. At day 7, the medium was replaced with cardiomyocyte maintenance media (RPMI‐1640 supplemented with B27 with insulin). Thereafter, cardiomyocyte maintenance media were replaced every 4 days. For the atrial differentiation protocol, retinoic acid (RA) addition was first optimized in pilot studies (Figures S2 and S3) and determined to be 0.75 μM RA every 24 hours from days 4 to 6.

### 2.3 Flow cytometry

hiPSC‐aCMs and hiPSC‐vCMs at day 20 to 30 postdifferentiation were dissociated into single cells as described in the Supplemental Information. The harvested cells were fixed in 4.1% PFA solution for 25 minutes and then washed and permeabilized in Saponin/FBS. Cells were subsequently incubated overnight in primary mouse‐cTnT (1:2000) and rabbit‐MLC2V (1:1000) antibodies. Subsequently, the cells were washed and incubated in secondary goat anti mouse Alexa‐488 (1:500) and goat anti rabbit Alexa‐647 (1:2000) antibodies for 1 hour, respectively. Cells were then washed and suspended in PBS for analysis. All analyses were performed using the BDJAZZ Fluorescence Activated Cell Sorter.

### 2.4 mRNA expression profiling

Gene expression profiling was conducted using multiplexed NanoString and real time quantitative PCR (qPCR). Pooled total RNA was used in both assays. The extracted RNA was reverse transcribed into cDNA which was used in the qPCR assay. Oligonucleotide sequences are described in Table S7. The multiplexed mRNA profiling was conducted using NanoString Technologies (Seattle, Washington) platform with a custom Codeset containing 250 gene probes. Analysis was performed on the Sprint instrument and nSolver analysis software with the Advanced Analysis module.

### 2.5 Atrial natriuretic peptide measurement

The levels of atrial natriuretic peptide (ANP) of hiPSC‐aCMs and ‐vCMs were measured by a competitive enzyme‐linked immunosorbent assay (ELISA) using a commercially available kit (Invitrogen, California). The assay was conducted according to the manufacturer's protocol and was measured using a spectrophotometric plate reader.

### 2.6 Cardiomyocyte enrichment

For cardiac enrichment, hiPSC‐aCMs and ‐vCMs at day 20 to 30 postdifferentiation were dissociated into single cells which were then enriched using a MidiMACS PSC‐derived Cardiomyocyte Isolation Kit (Miltenyi Biotec, Germany) according to the manufacturer's protocol. Enriched hiPSC‐CMs were seeded on Matrigel‐coated 24‐well plates at a seeding density of 600 000 cells per well.

### 2.7 Patch‐clamp recordings

Single hiPSC‐aCMs and hiPSC‐vCMs were plated on gelatin (0.1%) and Geltrex (1:10) at 30 000 cells per well. After 48 hours in culture, glass electrodes were used to achieve the whole‐cell configuration with single hiPSC‐CMs and only cells with gigaohm seals were used for further analysis. The formulation for internal and external recordings solutions are outlined in the Supplemental Information. Current recordings were performed using the Axon Instruments 700B amplifier and digitized at 20 kHz. All recordings were performed at 33‐35°C as maintained. For pacing at 1 Hz, gradually increasing amounts of current were injected with a 1 ms pulse width until reliable action potentials (APs) were triggered. The maximal upstroke velocity was determined by calculating the maximum derivative and the resting membrane potential was measured during a 5 second epoch without spontaneous activity 1 minute after break‐in. Further details on data analysis are found in the Supplemental Information.

### 2.8 Optical mapping

Optical mapping recordings were performed on enriched monolayers of hiPSC‐aCMs and ‐vCMs cultured in a 24‐well plate format at day 45 to 60 postdifferentiation. Imaging experiments were conducted using Ca^2+^ Tyrode's solution (formulation found in Supplemental Information). The hiPSC‐CMs were loaded with RH‐237, blebbistatin, and Rhod‐2AM sequentially before imaging as described.^12^, ^13^ Both RH‐237 and Rhod‐2AM were excited by 530 nm LEDs. Images were acquired at a frame rate of 100 frames/second by a sCMOS camera (Orca Flash 4.0V2, Hamamatsu Photonics, Japan) equipped with an optical splitter. The cells were paced using programmable stimulation. Data collection, image processing, and initial data analysis were accomplished using custom software. The multiwell optical mapping system was custom engineered in the lab based on a system as described previously.^12^, ^13^ Further details are found in the Supplemental Information.

### 2.9 Pharmacological analyses

The drugs used in this study are listed Table S8. Drug stocks were further diluted in Ca^2+^ Tyrode's solution prior to pharmacological testing with the final DMSO concentration in the experimental solution not exceeding 0.03% (v/v). Drug effects were studied in serum‐free conditions (ie, Ca^2+^ Tyrode's and drug only) at four doses by sequentially increasing the drug concentration in the same well with recordings at 20‐minute intervals.

### 2.10 Statistical analysis

Further details on data and statistical analysis can be found in the Supplemental Information. Unpaired *t* tests were conducted to compare two groups (ie, hiPSC‐aCMs vs hiPSC‐vCMs) in the analysis of qPCR, ELISA, patch clamp recordings, and optical mapping (baseline condition and normalized drug effects). Analysis of dose‐dependent effects was performed using one‐way ANOVA and Dunnett's post hoc test. All data are presented as mean ± SEM unless noted otherwise. Significance level for all statistical analysis was set at *P* < .05 with the following notation: *P < .05, **P < .01, ***P < .001.

## 3 RESULTS

### 3.1 RA treatment drives cardiac differentiation into atrial phenotype

**FIGURE 1:**
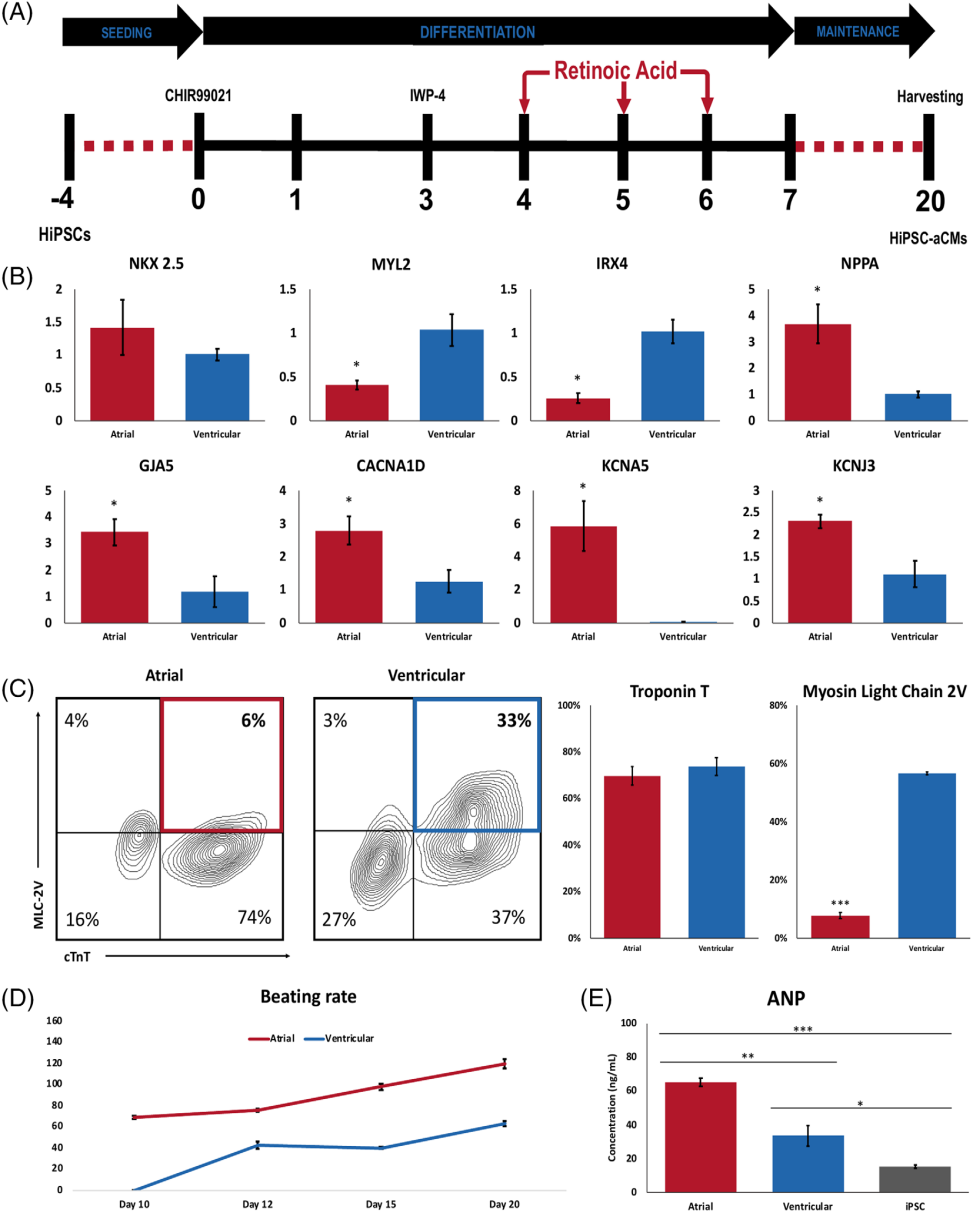
Directed differentiation of hiPSC‐derived atrial and ventricular CMs. A, Schematic depicting the atrial differentiation protocol. Doses of 0.75 μM retinoic acid (RA) were added to the cells every 24 hours on days 4, 5, and 6 with media exchanged to RPMI1640 + B27 with insulin at day 7. Cells were harvested for analysis at day 20. B, qPCR analysis of ventricular markers *MYL2* and IRX4, cardiac marker *NKX2.5*, and atrial markers *NPPA*, *GJA5*, *CACNA1D*, *KCNA5*, and *KCNJ3*. n = 3, unpaired *t* test, *P < .05. C, Flow cytometric analysis of cardiac troponin T (cTnT) and myosin light chain 2v (normalized to cTnT expression) in hiPSC‐aCMs and ‐vCMs. n = 4, unpaired *t* test, ***P < .001. D, Average beating rates of hiPSC‐aCMs and ‐vCMs from the day they begin to beat until day 20. n = 4 independent differentiation batches. E, Atrial Natriuretic peptide (ANP) concentration between hiPSCs, and hiPSC‐aCMs and ‐vCMs determined by competitive ELISA. n = 3 and n = 2 hiPSC lines, unpaired *t* test *P < .05, **P < .01, ***P < .001. Data are presented as mean ± SEM; One n represents one independent differentiation batch

We first optimized the atrial differentiation protocol by altering the concentration and timing of retinoic acid (RA) based on the molecular signatures of atrial phenotype as measured by qPCR and flow cytometry (Figures S2 and S3). Higher dose of RA reduced cardiac differentiation efficacy defined by the decrease in the cTnT^+^ proportion of the total cell population as measured by flow cytometry (Figure S2A). The finalized protocol to generate hiPSC‐aCMs included RA addition at 0.75 μM every 24 hours on days 4, 5, and 6 (Figure 1A) which was found as a balance between sufficiently driving atrial differentiation as defined by decreased ventricular marker myosin light chain 2 ‐ ventricular paralog (MLC‐2v) while having no impact cardiac differentiation efficacy Figures S2 and S3).

Compared with hiPSC‐vCMs, hiPSC‐aCMs were found to have no significant difference in pan cardiac phenotype. Expression of the pan cardiac transcript *NKX 2.5* measured by qPCR was similar between hiPSC‐aCMs and ‐vCMs (Figure 1B), as was cardiac troponin T (cTnT) protein expression measured by flow cytometry (Figures 1C and S1). The protein expression of MLC‐2v was reduced in hiPSC‐aCMs compared with hiPSC‐vCMs (8.0% ± 1.1% vs 57.0% ± 0.5%; *P* < .05) (Figure 1C). Furthermore, hiPSC‐aCMs displayed higher concentrations (increased by 91%) of atrial natriuretic peptide (ANP) at 65 ± 2 compared with 34 ± 6 ng/mL in hiPSC‐vCMs as measured by ELISA (*P* < .05).

The qPCR assay revealed that atrial‐specific transcripts such as atrial natriuretic peptide (*NPPA*), connexin 40 (*GJA5*), the calcium channel Ca_V_1.3 (*CACNA1D*), and the K^+^ channels K_v_1.5 (*KCNA5*) and K_ir_3.1 (*KCNJ3)* transcripts were all expressed at a significantly higher levels in hiPSC‐aCMs compared with hiPSC‐vCMs (*P* < .05, Figure 1B). Another ventricular marker, *IRX4*, also had decreased expression in hiPSC‐aCMs (Figure 1B). Furthermore, consistent with previous studies,^8^, ^9^, ^10^, ^14^, ^15^ hiPSC‐aCMs started beating at day 10 or earlier and exhibited an increased beating frequency relative to hiPSC‐vCMs, which started beating around day 10 to 12 postdifferentiation.

### 3.2 Gene expression analysis of hiPSC‐aCMs

**FIGURE 2:**
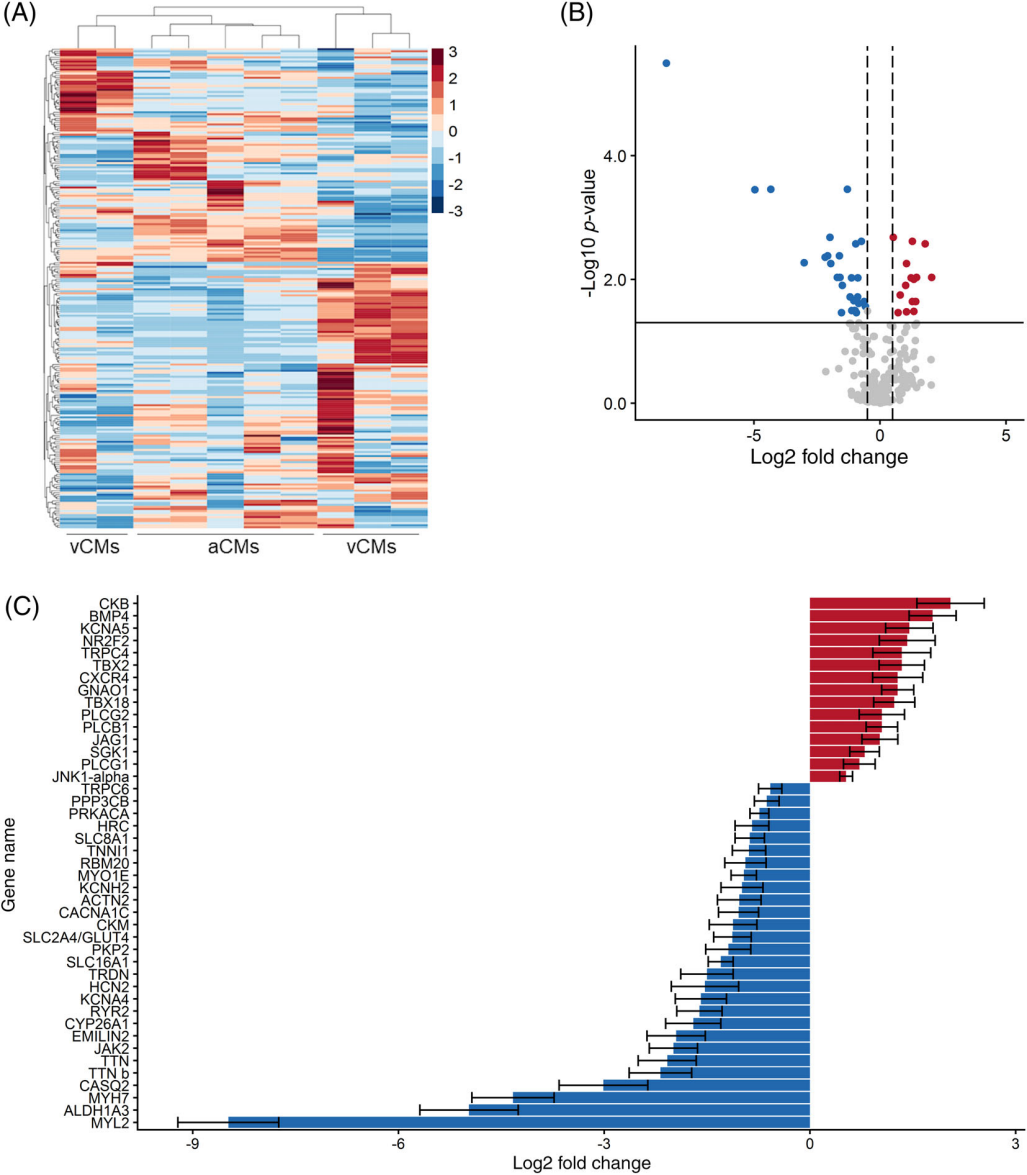
Gene expression analysis of hiPSC‐aCMs and ‐vCMs using NanoString. Global gene expression pattern of hiPSC‐aCMs and ‐vCMs shown in A, heat map of the expression of the 250 genes across samples of hiPSC‐aCMs and ‐vCMs. The cluster dendrogram shows the unsupervised hierarchical clustering that was conducted using the agglomerative algorithm and the Euclidian distance criterion. B, Differentially expressed genes between hiPSC‐aCMs and ‐vCMs expressed in volcano plot shows 14 upregulated (red) and 27 downregulated (blue) genes in hiPSC‐aCMs. Solid horizontal line represents the Benjamini‐Hochberg false discovery rate (FDR) adjusted *P*‐value <.05 (−log10 = 1.3). Dashed vertical lines represent the arbitrary log2 fold change cut‐off of −0.5 and 0.5. C, Forty‐two differentially expressed genes identified from the statistical criteria of FDR adjusted *P*‐value <.05 and log2 fold change of <−0.5 and >0.5. Data are presented as mean ± SEM. n = 5 independent differentiation batches

We performed an extensive gene expression analysis of hiPSC‐aCMs and hiPSC‐vCMs using NanoString technology in which each mRNA copy was digitally counted for accurate and sensitive detection of gene expression.^16^ Five independent differentiation batches of each cardiac subtype were included in the analysis. The unsupervised hierarchical clustering analysis showed clear grouping of hiPSC‐aCM samples that were segregated relative to hiPSC‐vCMs (Figure 2A). The gene expression profile of the hiPSC‐vCM samples were more variable with 2 samples closer in distance to the hiPSC‐aCMs while three samples displayed clear segregation (Figure 2A). The overall difference in global gene expression and lineage between hiPSC‐aCMs and ‐vCMs was also captured in the principal component analysis (PCA, Figure S4A). Out of the 250 transcripts analyzed, 200 genes were detected above background noise defined by a threshold of 50 raw digital counts as determined by the negative controls of the assay. In the hiPSC‐aCMs, 14 and 27 genes were significantly upregulated and downregulated, respectively (Figure 2C). As expected, hiPSC‐aCMs displayed significantly higher expression profiles of atrial‐specific markers including atrial‐specific K^+^ channel K_v_1.5 (*KCNA5*) and transcription factors (*NR2F2* and *TBX18*) (Figure 2C). Meanwhile, hiPSC‐vCMs displayed higher expression of ventricular‐specific genes such as those encoding for contractile proteins *MYL2*, *MYH7*, and the L‐type Ca^2+^ channel isoform Ca_v_1.2 (*CACNA1C*) (Figure 2C). The genes encoding for the proteins in the sarcoplasmic reticulum complex such as *TRDN*, *CASQ2*, and *RYR2* were expressed in significantly lower amounts in the hiPSC‐aCMs samples (Figure 2C). Meanwhile, pan‐cardiac markers *NKX2‐5* and *TNNT2* were expressed at similar levels in both hiPSC‐aCMs and ‐vCMs, further corroborating the efficiency of the differentiation protocol (Figure S4B).

### 3.3 Functional phenotyping of hiPSC‐derived atrial cardiomyocytes

**FIGURE 3:**
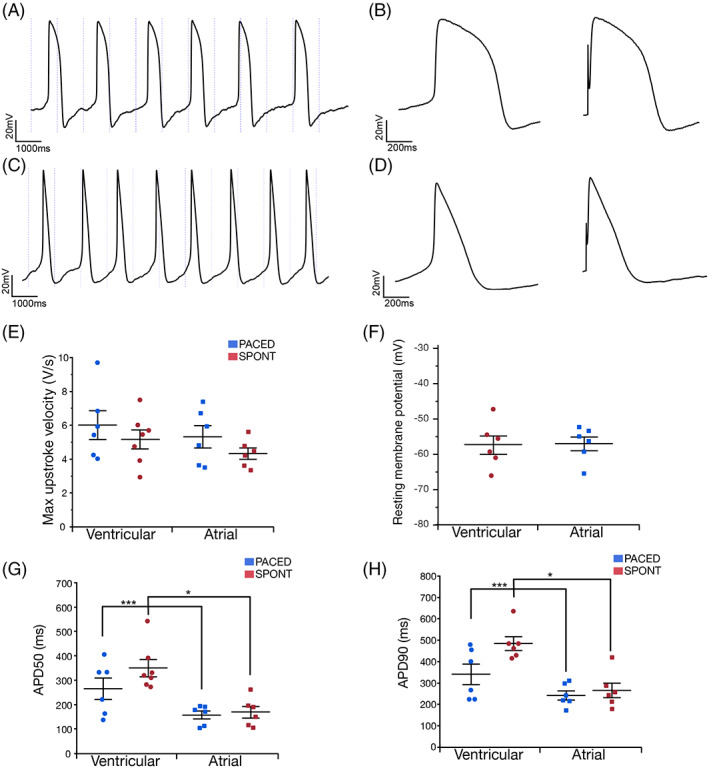
hiPSC‐aCMs and ‐vCMs have distinct electrophysiological characteristics. Single differentiated hiPSC‐aCMs and ‐vCMs were plated on gelatin and Geltrex after 30 days in culture. A, Whole cell current clamp recordings from a spontaneously beating hiPSC‐vCM. B, Recorded action potential (APs) demonstrates typical prolonged plateau phase in both spontaneous (left) and/or paced at 1 Hz (right). C, Current clamp recording from a spontaneously beating hiPSC‐aCM. D, Single AP from hiPSC‐aCM demonstrates shortened action potential duration (APD) and lack of prolonged plateau phase, spontaneous (left), paced at 1 Hz (right). E, The first differential of voltage recordings from hiPSC‐aCMs and ‐vCMs were used to calculate the maximal upstroke velocities. F, One minute after achieving the whole‐cell configuration, the average resting membrane potential was measured. G, Spontaneously beating and 1 Hz paced APs were assessed for duration at 50% of peak (APD_50_), and H, 90% of peak (APD_90_). Statistics were performed by unpaired *t* test. *P < .05, ***P < .005. Data are presented as mean ± SEM. Two differentiation batches were included in this analysis

We compared the electrophysiological characteristics of the differentiated hiPSC‐aCMs and ‐vCMs using whole‐cell patch clamp. Confirming our observations in tissue culture, the spontaneous beating rates were higher in the single hiPSC‐aCMs than in ‐vCMs (Figure 3A,C). Whole cell current clamp recordings demonstrated the ventricular‐like AP morphology of hiPSC‐vCMs with a clear and prolonged plateau phase while the AP of the hiPSC‐aCMs displayed atrial‐like morphology with a shorter action potential duration (APD) and a lack of prolonged plateau phase at both spontaneous beating rates (Figure 3B,D, left panel) and paced at 1 Hz (Figure 3B,D, right panel). No statistical differences were observed in the resting membrane potential and the maximum upstroke velocity of hiPSC‐aCMs and ‐vCMs. The APD at 50% (APD_50_) and 90% (APD_90_) of the peak voltage were significantly shorter in hiPSC‐aCMs than ‐vCMs at both spontaneous beating rates (APD_50_: 157 ± 16 vs 349 ± 35 ms, *P* < .005; APD_90_: 249 ± 34 vs 484 ± 30 ms, *P* < .005) and paced at 1 Hz (APD_50_: 157 ± 16 vs 264 ± 44 ms, *P* < .05; APD_90_: 242 ± 22 vs 341 ± 48 ms, *P* < .05).

**FIGURE 4:**
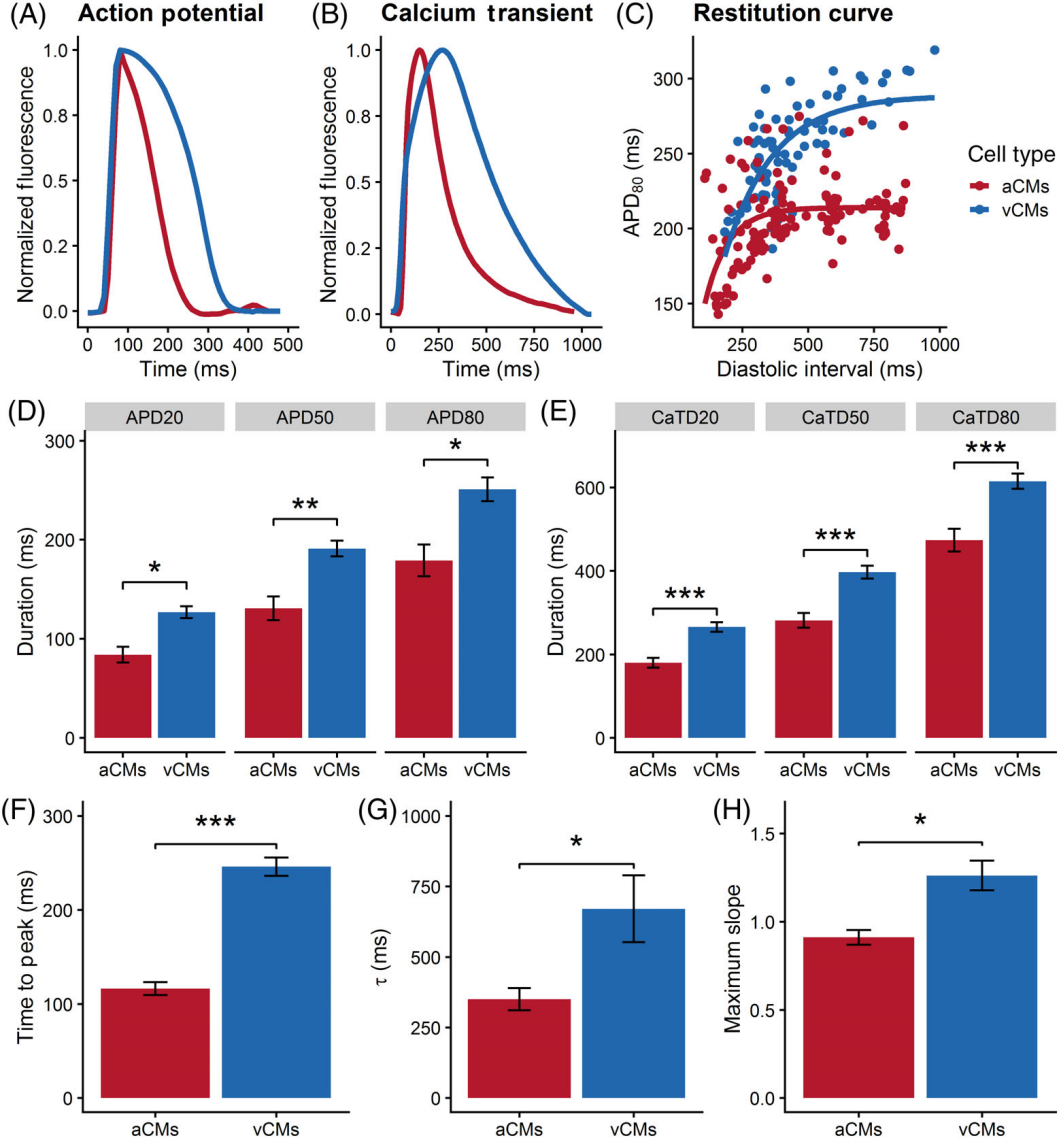
Functional phenotyping of hiPSC‐derived atrial and ventricular CMs using optical mapping. Representative average traces of A, action potential and B, Ca^2+^ transients of hiPSC‐aCMs and ‐vCMs electrically paced at 1 Hz. C, Electrical restitution curve measured at APD_80_ relative to the diastolic interval (DI). D, Quantification of early‐ (APD_20_), mid‐ (APD_50_), and late‐ (APD_80_) repolarization, unpaired *t* test, *P < .05, **P < .01. E, Quantification of early‐ (CaTD_20_), mid‐ (CaTD_50_), and late‐ (CaTD_80_) Ca^2+^ transient decay, unpaired *t* test, ***P < .001. F, Time to peak (TTP) of the Ca^2+^ transient, unpaired *t* test, ***P < .001. G, Time constant (τ) of Ca^2+^ decay, unpaired *t* test *P < .05. H, Maximum slope of the electrical restitution as shown in panel C, unpaired *t* test, *P < .05. Electrical restitution curves were measured under a variable rate pacing protocol (60‐200 bpm) as described in the Supplemental Information. n = 4 (four independent differentiation batches) and cardiac enriched hiPSC‐aCMs and ‐vCMs were analyzed in these set of experiments. Data are presented as mean ± SEM

We further assessed the functional properties of hiPSC‐aCMs and hiPSC‐vCMs using optical mapping with simultaneous measurement of APs and calcium transients (CaT). Like the patch clamp recordings, optical membrane voltage measurements revealed similar atrial‐like and ventricular‐like AP morphology in the hiPSC‐aCMs and ‐vCMs, respectively (Figure 4A). AP and CaT durations were quantified at early, mid, and late repolarization (APD_20_, APD_50_, and APD_80_) and Ca^2+^ decay (CaTD_20_, CaTD_50_, and CaTD_80_), respectively. These stages reflect different phases of ionic currents across the plasma membrane and the extrusion of Ca^2+^ handling mechanics.

For these experiments, both hiPSC‐aCMs and hiPSC‐vCMs were paced at 1 Hz. All measured levels of the APD were significantly shorter in hiPSC‐aCMs compared with hiPSC‐vCMs (APD_20_: 84 ± 8 vs 127 ± 6 ms, *P* < .05; APD_50_: 131 ± 12 vs 191 ± 8 ms, *P* < .01; APD_80_: 179 ± 16 vs 251 ± 12 ms, *P* < .05; Figure 4D). The overall CaTD of hiPSC‐aCMs was significantly shorter than that of hiPSC‐vCMs (CaTD_20_: 180 ± 12 vs 266 ± 12 ms, *P* < .001; CaTD_50_: 282 ± 18 vs 397 ± 16 ms, *P* < .001; CaTD_80_: 474 ± 27 vs 615 ± 18 ms, *P* < .001; Figure 4E). Compared with hiPSC‐vCMs, hiPSC‐aCMs displayed significantly faster CaT time‐to‐peak (hiPSC‐aCMs: 116 ± 7 ms vs hiPSC‐vCMs: 246 ± 10 ms, *P* < .05) and faster decay kinetics (τ; hiPSC‐aCMs: 350 ± 39 ms vs hiPSC‐vCMs: 671 ± 118 ms, *P* < .05) indicating that Ca^2+^ handling mechanics are accelerated in hiPSC‐aCMs (Figure 4F,G).

The direct comparison between whole‐cell patch clamp and optical mapping read‐outs paced at 1 Hz is shown in Figure S7. We observed no differences in the read‐outs of hiPSC‐aCMs at APD_20_ (optical: 84 ± 8 ms, patch: 98 ± 12 ms) and APD_50_ (optical: 131 ± 12 ms, patch: 169 ± 19 ms). However, APD_80_ of hiPSC‐aCMs measured by patch clamp was longer than the optical APD_80_ (253 ± 22 vs 179 ± 16 ms, *P* < .05). Similarly, both APD_20_ (216 ± 22 vs 127 ± 6 ms) and APD_80_ (393 ± 62 vs 251 ± 12 ms) of hiPSC‐vCMs measured by patch clamp were longer than the comparable optical measurements. APD_50_ of hiPSC‐vCMs did not show a statistical difference between the two assays (optical: 191 ± 8 ms, patch: 308 ± 60 ms).

Rate‐dependent properties are critical in cardiac function. A variable rate protocol (Figure S6) in which the hiPSC‐CMs were electrically paced with increasing frequency at every cycle was used to investigate the electrical restitution dynamics. The electrical restitution curve reflects the ability of the cardiac system to accommodate a higher pacing rate by progressive shortening of APD_80_ and is described as APD_80_ in relation to the diastolic interval (DI). Compared with hiPSC‐vCMs, the electrical restitution curve of the hiPSC‐aCMs displayed a flatter portion and did not show APD_80_ shortening at longer diastolic intervals (Figure 4F). The extensive shortening in APD_80_ started at shorter diastolic intervals for hiPSC‐aCMs (<275 ms) compared with hiPSC‐vCMs (<500 ms). The maximum slope of the restitution curve was higher in hiPSC‐vCMs compared with hiPSC‐aCMs (1.26 ± 0.08 vs 0.91 ± 0.04, *P* < .05; Figure 4G) indicating faster kinetics of APD in response to higher pacing rate.

### 3.4 In vitro screening for atrial‐selective pharmacology

**FIGURE 5:**
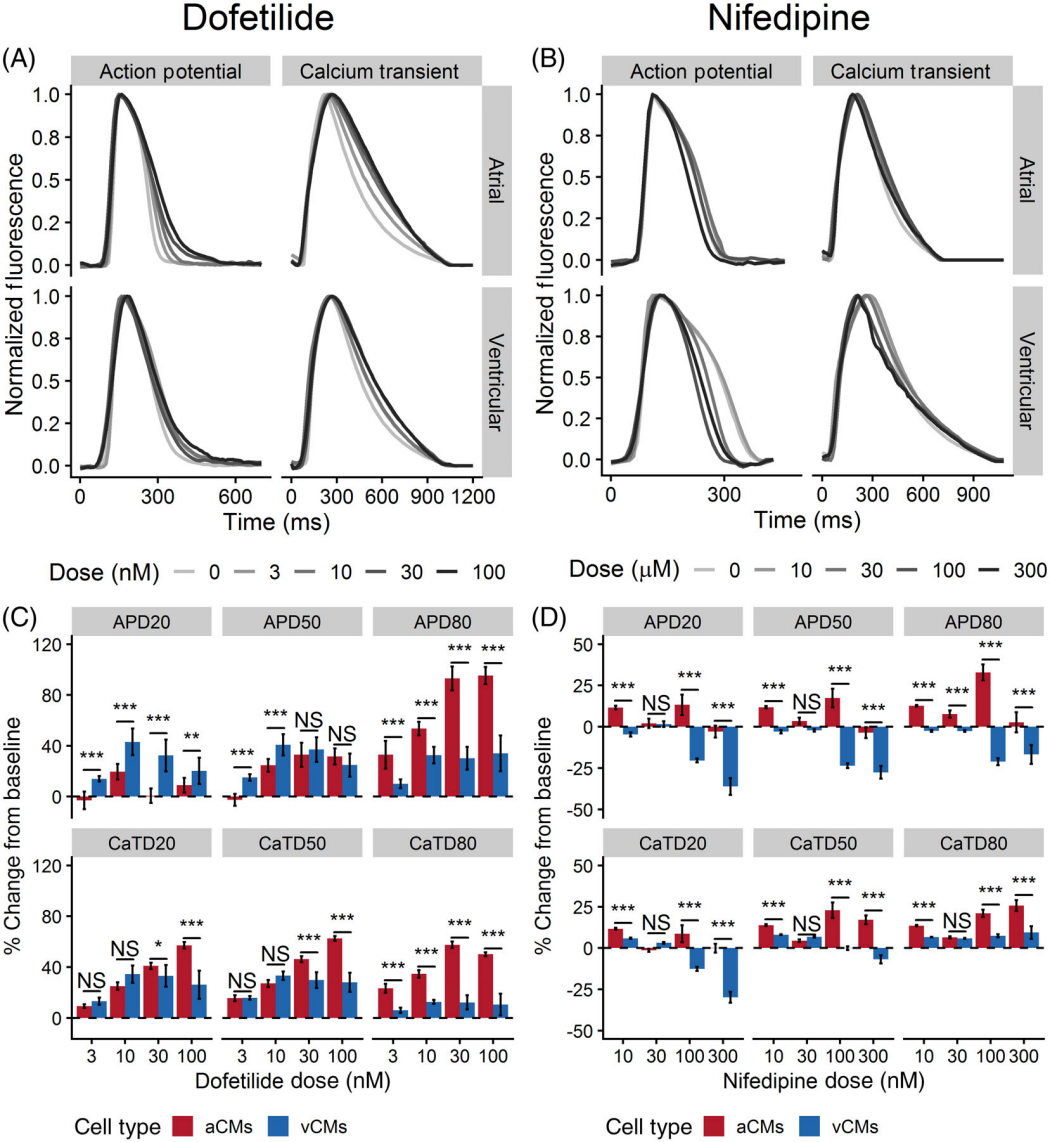
The effects of dofetilide and nifedipine on action potential and Ca^2+^ transient of hiPSC‐aCMs and ‐vCMs. Representative traces of action potential and Ca^2+^ transients illustrating the effects of A, dofetilide and B, nifedipine on hiPSC‐aCMs and ‐vCMs. Higher drug doses are presented by a progressively darker shade. The effects of C, 4‐aminopyridine and D, nifedipine on normalized (percent change from predrug baseline) action potential duration (APD) and Ca^2+^ transient duration (CaTD); both parameters being measured at 20%, 50%, and 80%. Dashed line is the normalized predrug control presented as 0% change. n = 6 from six independent differentiation batches. hiPSC‐derived atrial cardiomyocytes (aCMs) are shown in red while hiPSC‐derived ventricular cardiomyocytes (vCMs) are presented in blue. Data are presented as mean ± SEM. Drug effects were compared between hiPSC‐aCMs and ‐vCMs at each dose using unpaired *t* test, *P < .05, **P < .001, ***P < .001. NS stands for not significant

We first established the utility of optical mapping to detect a pan‐cardiac pharmacological response by using dofetilide, a strong blocker of the rapid delayed rectifier K^+^ current (I_Kr_),^17^ an ionic current expected to be present in both hiPSC‐aCMs and ‐vCMs.^18^ Dofetilide elicited a dose‐dependent response in both hiPSC‐aCMs and ‐vCMs. Compared with predrug baseline, dofetilide at 100 nM prolonged APD_80_ of both hiPSC‐aCMs from 182 ± 16 ms to 355 ± 24 ms (95% ± 7% prolongation) and of hiPSC‐vCMs from 238 ± 20 ms to 319 ± 45 ms (34% ± 14% prolongation, *P* < .05; Table S1 and Figure 5C). The drug prolonged early‐repolarization (APD_20_) of hiPSC‐vCMs at 10 and 30 nM while having no effect on APD_20_ of hiPSC‐aCMs at all tested doses (Table S1). Additionally, CaTD_50_ and CaTD_80_ of both hiPSC‐aCMs and ‐vCMs were significantly prolonged in response to dofetilide (Table S1). However, hiPSC‐aCMs appeared to be more sensitive to dofetilide as the APD_80_ was significantly prolonged at the lowest tested dose of 3 nM (from 182 ± 26 to 241 ± 26 ms, *P* < .05; Table S1) and displayed a larger dose‐response (Figure S8).

Next, we demonstrated the functional differences in the ion channel profiles of hiPSC‐aCMs and ‐vCMs. We aimed to show that the ultrarapid outward current (I_Kur_) produced by the channel K_v_1.5 (*KCNA5*) was functional and specific to hiPSC‐aCMs, while the inward Ca^2+^ current (I_Ca,L_) produced the voltage‐dependent L‐type Ca^2+^ channel Ca_V_1.2 (*CACNA1C*) was functional and specific to hiPSC‐vCMs. We used two relatively selective compounds, 4‐aminopyridine (4AP) and nifedipine, to dissect the presence of functional I_Kur_ and I_CaL_, respectively. While nifedipine is also known to block Cav1.3, it is expected to have a preferential effect at lower concentrations on Ca_V_1.2 based on the literature which indicates ~13‐fold higher block on Ca_V_1.2 than Ca_V_1.3.^19^

At the highest tested dose (300 nM), nifedipine significantly decreased APD_50_ of hiPSC‐vCMs from 170 ± 14 to 121 ± 16 ms (28% ± 4% shortening) and decreased CaTD_50_ from 357 ± 10 to 333 ± 23 ms (30% ± 3% shortening) (Figure 5D; Table S2). We observed a trend in APD_50_ shortening of hiPSC‐aCMs in response to increasing the nifedipine dose, but the drug elicited a significantly stronger dose‐dependent shortening in both APD and CaTD of hiPSC‐vCMs compared with hiPSC‐aCMs (Figures S8 and S9). Observing the percent change from predrug control, nifedipine induced differential response in overall APD and CaTD between hiPSC‐aCMs and ‐vCMs at 10, 100, and 300 nM (Figure 5D).

**FIGURE 6:**
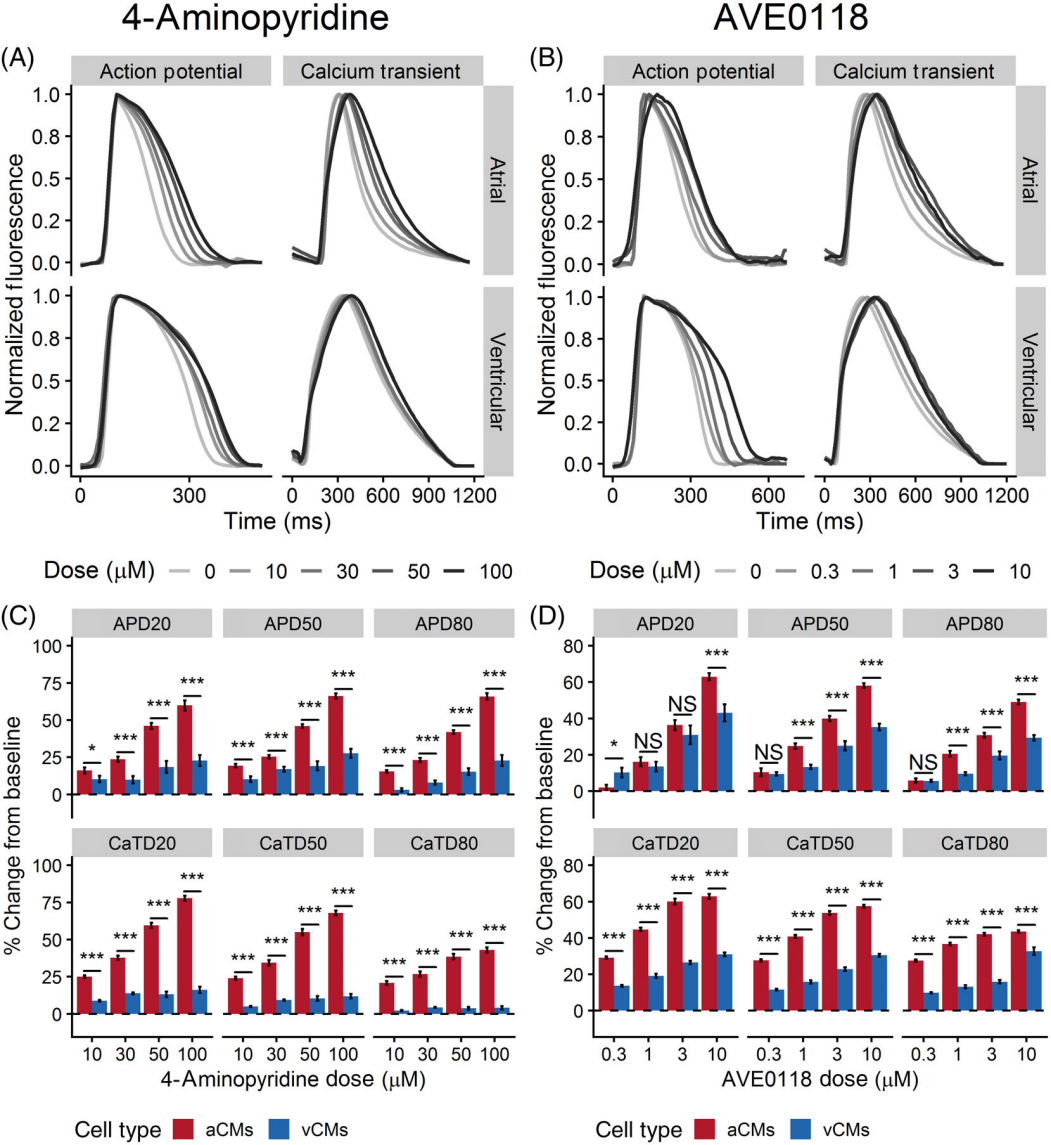
The effects of 4‐aminopyridine (4AP) and AVE0118 on action potential and Ca^2+^ transient of hiPSC‐aCMs and ‐vCMs. Representative traces of action potential and Ca^2+^ transients illustrating the effects of A 4‐aminopyridine (4AP) and B, AVE0118 on hiPSC‐aCMs and ‐vCMs. Higher drug dose is presented by a progressively darker shade. The effects of C dofetilide and D, vernakalant on normalized (percent change from predrug baseline) action potential duration (APD), and B, Ca^2+^ transient duration (CaTD); both parameters being measured at 20%, 50%, and 80%. Dashed line is the normalized predrug control presented as 0% change. n = 6 from six independent differentiation batches. hiPSC‐derived atrial cardiomyocytes (aCMs) are shown in red while hiPSC‐derived ventricular cardiomyocytes (vCMs) are presented in blue. Data are presented as mean ± SEM. Drug effects were compared between hiPSC‐aCMs and ‐vCMs at each dose using unpaired *t* test, *P < .05, **P < .001, ***P < .001. NS stands for not significant

In hiPSC‐aCMs, 4AP prolonged APD and CaTD in a dose‐dependent manner with a statistically significant change starting at 30 μM (Figure 6A,C; Table S3). 4AP significantly prolonged early‐repolarization (APD_20_) of hiPSC‐aCMs by 46% ± 2% and 66% ± 2% at 50 and 100 μM, respectively (APD_20_ at baseline: 82 ± 8, at 50 μM: 120 ± 9 ms, at 100 μM: 131 ± 9 ms, *P* < .05) (Figure 6C and Table S3). In contrast, 4AP prolonged APD_20_ of hiPSC‐vCMs by 23% ± 4% (APD_20_ at baseline: 138 ± 8 ms, at 100 μM: 170 ± 9 ms) at the highest tested dose of 100 μM (Figure 6C and Table S3). hiPSC‐aCMs showed greater change in APD to relative to predrug control at all concentrations of 4AP compared with hiPSC‐vCMs (Table S3), This is corroborated by the steeper trend of the dose response relationship in hiPSC‐aCMs (Figure S8). Additionally, the overall CaTD of hiPSC‐aCMs were prolonged after exposure to 4AP at 10 μM while the drug had a significant effect on CaTD of hiPSC‐vCMs at 30 μM (CaTD_50_ elongation from baseline: 68% ± 2% vs 12% ± 2%, *P* < .05) (Table S3).

We then demonstrated the effectiveness of our drug screening platform in assessing the effects of experimental compounds designed to have targeted effects on atrial‐specific ion channels using AVE0118 and UCL1684.

AVE0118 is an experimental drug that blocks I_Kur_, the G‐protein‐activated K^+^ current (I_KAch_)_,_ and the transient outward K^+^ current (I_to_) at a similar dose range.^20^ Both I_Kur_ and I_KAch_ are atrial‐specific ionic currents. AVE0118 prolonged mid‐ and late‐ repolarization (APD_50_ and APD_80_) of both hiPSC‐aCMs and ‐vCMs at the two highest tested doses (3 and 10 μM; Table S4). Similarly, AVE0118 had significant effects on CaTD_50_ and CaTD_80_ of hiPSC‐aCMs and ‐vCMs at all tested doses (Table S4). However, the APD_50_ and APD_80_ of hiPSC‐aCMs were significantly prolonged at a lower dose of 1 μM (control: 200 ± 14 ms, 1 μM: 244 ± 16 ms; Table S4). Furthermore, the atrial‐selective effects of the drug were demonstrated by a larger proportional prolongation in APD_50_ and APD_80_ of hiPSC‐aCMs compared with hiPSC‐vCMs at 1, 3, and 10 μM (APD; Figure 6D). Furthermore, AVE0118 induced a larger proportional prolongation in CaTD of hiPSC‐aCMs compared with hiPSC‐vCMs at all tested doses (Figure 6D). Early repolarization (APD_20_) of hiPSC‐aCMs also displayed a large dose‐dependent response (Figure S8) with a proportionally larger prolongation at 10 μM (63% ± 2% vs 43% ± 5%, *P* < .05; Figure 6D).

**FIGURE 7:**
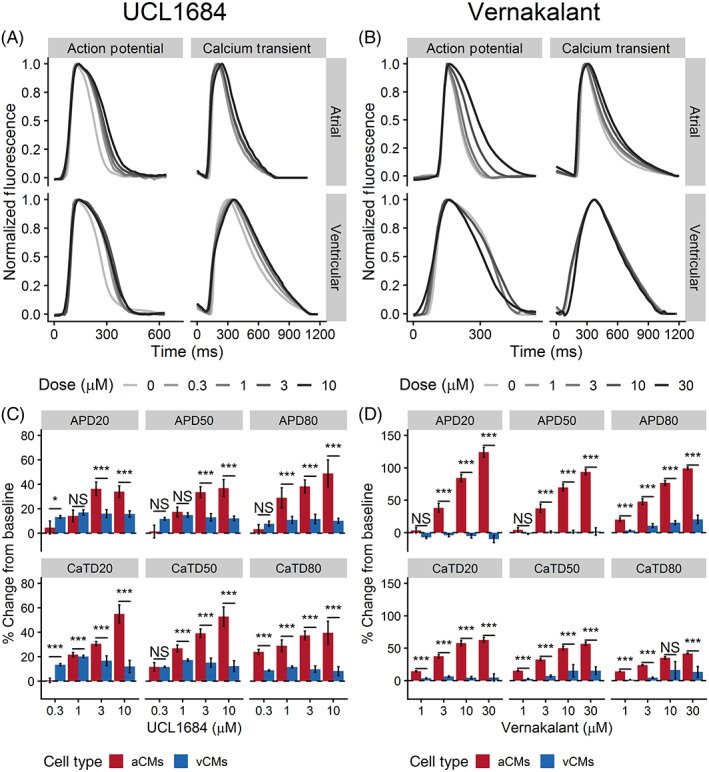
The effects of UCL1684 and vernakalant on action potential and Ca^2+^ transient of hiPSC‐aCMs and ‐vCMs. Representative V_m_ and Ca^2+^ transients illustrating the effects of A, UCL1684 and B, vernakalant on hiPSC‐aCMs and ‐vCMs. Higher drug doses are presented by a progressively darker shade. The effects of C, AVE0118 and D, UCL1684 on normalized (percent change from predrug baseline) action potential duration (APD) and Ca^2+^ transient duration (CaTD); both parameters being measured at 20%, 50%, and 80%. Dashed line is the normalized predrug control presented as 0% change. n = 6 from six independent differentiation batches. hiPSC‐derived atrial cardiomyocytes (aCMs) are shown in red while hiPSC‐derived ventricular cardiomyocytes (vCMs) are presented in blue. Data are presented as mean ± SEM. Drug effects were compared between hiPSC‐aCMs and ‐vCMs using unpaired *t* test at each dose, *P < .05, **P < .001, ***P < .001. NS stands for not significant

UCL1684 is purported to be a potent direct pore blocker of the small conductance Ca^2+^ activated K^+^ channel (SK channel)^21^ and was expected to induce a dose‐dependent atrial‐selective response. In hiPSC‐aCMs, UCL1684 treatment resulted in a significantly prolonged APD_80_ at 3 and 10 μM (from predrug control: 136 ± 11 ms to 3 μM: 188 ± 25 ms or 38% ± 5% prolongation, and to 10 μM: 206 ± 32 ms or 49% ± 11% prolongation, *P* < .05; Figure 7C and Table S5). UCL1684 prolonged CaTD_80_ of hiPSC‐aCMs at all tested doses (baseline: 300 ± 15 ms, at 0.3 μM: 372 ± 23 ms, at 1 μM: 387 ± 33 ms, at 3 μM: 413 ± 24 ms, at 10 μM: 416 ± 39 ms, *P* < .05; Table S5). In contrast, UCL1684 exposure showed no statistically significant effect on overall APD and CaTD of hiPSC‐vCMs. The sensitivity of hiPSC‐aCMs to UCL1684 was also reflected in the dose‐response relationship showing a prolongation APD_80_, in contrast to the minimal prolongation in APD_80_ of hiPSC‐vCMs (Figure S8).

Finally, we tested the effects of vernakalant which is a multi‐ion channel blocker that blocks the fast and late inward Na^+^ current (I_Na_, I_NaL_, respectively), the I_Kur_, and the I_KAch._ ^22^ The drug is used clinically for intravenous cardioversion of patients in AF^23^ and was expected to induce an atrial‐specific effect due to its I_Kur_ and I_KAch_ blocking properties.

Vernakalant elicited a positive dose‐dependent response in both APD and CaTD of hiPSC‐aCMs with minimal measurable effects on hiPSC‐vCMs (Table S6; Figures S8 and S9). Vernakalant demonstrated atrial‐selectivity with statistically significant differences between APD and CaTD of hiPSC‐aCMs and ‐vCMs at doses of 3, 10, and 30 μM (Figure 7D). Compared with APD at baseline, vernakalant at 10 μM significantly prolonged APD_20_, APD_50_, and APD_80_ of hiPSC‐aCMs by 84% ± 6%, 70% ± 5%, and 77% ± 4%, respectively (Figure 7D). Additionally, vernakalant at 10 μM prolonged CaTD_20_, CaTD_50_, CaTD_80_ of hiPSC‐aCMs by 58% ± 4%, 50% ± 3%, 35% ± 5%, respectively (Figure 7D). At clinically relevant concentrations (30 μM), vernakalant greatly affected early repolarization of hiPSC‐aCMs (APD_20_ prolonged by 124% ± 8%; Figure 7D). At 30 μM, vernakalant prolonged APD_80_ of hiPSC‐vCM by 20% ± 7% (APD_80_: 238 ± 22 ms at baseline vs 289 ± 30 ms at 30 μM, *P* < .05; Figure 7D and Table S6). Except for APD_80_ prolongation at 30 μM, vernakalant had no statistically significant effect on overall APD and CaTD of hiPSC‐vCMs at the lower doses (Table S6).

## 4 DISCUSSION

In this study, we were successful in efficiently differentiating hiPSCs into a monolayer of cardiomyocytes with an atrial phenotype by modifying the GiWi protocol.^11^ We used multiple phenotypic approaches such as qPCR, digital multiplexed gene expression analysis with NanoString technology, flow cytometry, ELISA, voltage measurements with current clamp electrophysiology as well as simultaneous voltage and Ca^2+^ transient measurements with optical mapping to demonstrate a clear and distinct atrial phenotype. Unique to our study, we completed an in‐depth pharmacological analysis with simultaneous voltage and Ca^2+^ measurements to demonstrate the differential responses of these chamber‐specific cardiomyocytes, and their utility as a translational model in screening for the safety and efficacy of novel atrial‐specific compounds for the treatment of AF.

Our observations support previous data in showing that atrial specification is in part mediated by RA.^6^, ^8^, ^9^, ^10^, ^16^ In our protocol, atrial differentiation was accomplished by adding 0.75 μM RA 24 hours after WNT inhibition, with a total exposure time of 72 hours. The generated hiPSC‐aCMs showed an atrial‐specific phenotype as validated at both protein and transcript levels with a decrease in ventricular‐specific and an increase in atrial‐specific markers. These results suggest that RA, at the dose and temporal exposure used in this study, maintains cardiac differentiation efficiency while pushing the differentiation process into an atrial lineage.

As a complementary assay, we used the NanoString digital multiplexed gene expression analysis to assess the expression of 250 genes custom‐curated from the existing literature. We found *MYL2* and *MYH7*, markers of the ventricular phenotype, to be significantly differentially expressed between hiPSC‐aCMs and ‐vCMs, matching the gene expression pattern of native adult human right atrial and left ventricular tissues.^24^ Another ventricular‐specific marker *KCNA4* ^25^ which encodes for the Kv1.4 channel of the slow I_to_ was downregulated in hiPSC‐aCMs. Canonical atrial markers such as *KCNA5* and *NR2F2* were also confirmed to be differentially upregulated in hiPSC‐aCMs. Other markers of human atrial specificity such *CXCR4*, *GNAO1*, *JAG1*, *PLCB1*, and *TBX18* as retrieved from the GTEx database^26^ were upregulated in our hiPSC‐aCMs further demonstrating the effect of RA on driving the differentiation pathway into an atrial lineage.

*MYL7*, thought to be an atrial‐specific marker, was not found to have a significantly higher expression in hiPSC‐aCMs. The differential expression of MLC‐2a may however require additional maturation of the hiPSC‐CMs. Other studies^11^, ^27^ have shown a high expression in MLC‐2a at day 20 postdifferentiation and a subsequent decrease over time in culture systems generating predominantly ventricular hiPSC‐CMs. One study has shown a higher expression of MLC‐2a in hiPSC‐aCMs analyzed at a later date (earliest at day 60).^6^

Electrophysiological differences between atrial and ventricular phenotypes, in terms of voltage and Ca^2+^ handling, define their function and are critical to the development and determination of efficacy of atrial‐specific compounds. As demonstrated by whole‐cell patch clamp and optical mapping measurements, the hiPSC‐aCMs generated in this study exhibited atrial‐like AP and Ca^2+^ handling properties. Namely, the AP of hiPSC‐aCMs was significantly shorter, along with a lack of a prolonged plateau phase as opposed to the AP of hiPSC‐vCMs, an observation that is aligned with native cardiomyocyte electrophysiology.^28^ Similarly, the CaT of hiPSC‐aCMs had faster kinetics with a faster decay time as reflected by the differential expression of Ca^2+^ channel isoforms, further demonstrating the differential physiology between hiPSC‐aCMs and ‐vCMs.

In terms of APD measurements, we observed a good correlation between the patch clamp and optical mapping recordings for hiPSC‐aCMs. In hiPSC‐vCMs, however, the optical AP measurements were shorter overall than patch clamp recordings. This discrepancy may be attributed to the heterogeneity of our current ventricular differentiation protocol which generated predominantly ventricular cardiomyocytes but also contain a small proportion of nonventricular phenotypes (ie, atrial myocytes and nodal cells). Thus, the optical AP signals represents an average from about 300 000 cells in each 1 cm^2^ region of interest.

Another hallmark of cardiomyocyte function is rate‐dependence, as described by the electrical restitution curve.^29^ We observed that the electrical restitution properties were different between hiPSC‐aCMs and ‐vCMs. Compared with hiPSC‐vCMs, hiPSC‐aCMs displayed a steady‐state‐like property by undergoing minimal APD_80_ shortening in response to the lower ranges of the pacing protocol (cycle lengths of about 400‐1000 ms) indicating full recovery of ion channel kinetics at these pacing ranges. In contrast, the hiPSC‐vCMs displayed consistent APD_80_ shortening at the same pacing range. It is important to note that APD restitution curves are likely different when using the standard steady‐state extra stimulus protocol compared with dynamic pacing, particularly in cardiomyocytes with immature Ca^2+^ handling and memory.^29^ In relation to dynamic pacing protocol, hiPSC‐vCMs have steeper maximum slope of the restitution curve compared with hiPSC‐aCMs as steady‐state APD is the principal determinant of the slope of the ventricular restitution curve.^30^

The presence of specific ion channel currents (ie, I_Kur_, I_KAch_, and I_CaL_) explain, in part, the functional differences between the two cardiac chamber subtypes, the expressions of which were already shown in our qPCR and NanoString assays. We used a series of compounds (4‐aminopyridine, dofetilide, vernakalant, AVE0118, UCL1684, and nifedipine) to demonstrate the function of atrial‐specific ionic currents in our model system and were able to show the expected chamber specific differences between hiPSC‐aCMs and ‐vCMs.

Dofetilide (DF) served as a positive control in our optical mapping assay as a clinically relevant drug which has a strong effect on I_Kr_ in both atria and ventricular CM.^31^ As expected, dofetilide affected the repolarization of both hiPSC‐aCMs and ‐vCMs, confirming the presence of I_Kr_ in both cell types. At clinically relevant doses of DF (3 and 10 nM), hiPSC‐aCMs displayed greater sensitivity to the drug indicating a larger proportional contribution of I_Kr_ in the AP of hiPSC‐aCMs relative to hiPSC‐vCMs. This may partly explain the effectiveness of the drug in the clinical treatment of AF. However, clinical use of the drug to treat AF is limited due to its tendency to induce QTc prolongation. This pro‐arrhythmic risk of TdP^32^ which was captured by the prolongation of APD_80_, an in vitro surrogate of QTc, in the hiPSC‐vCMs. This finding supports the utility of our optical mapping assay in predicting the risk of ventricular proarrhythmia in vitro.

The compound 4AP has been shown to selectively block K_v_1.4 (I_to_) and K_v_1.5 (I_Kur_)^33^ and is therefore expected to elicit a response in hiPSC‐aCMs at lower doses than in hiPSC‐vCMs as I_Kur_ (K_v_1.5) is a strong functional indicator of atrial phenotype. Confirmation of the atrial expression of I_Kur_ channels was demonstrated by the stronger dose‐dependent hiPSC‐aCM AP prolongation to 4AP at all tested doses (10, 30, 50, and 100 μM) suggesting selective sensitivity of hiPSC‐aCMs to 4AP due to a greater expression of K_v_1.5. The inhibitory effects of 4AP were observed at higher doses (50 and 100 μM) in hiPSC‐vCMs which can be attributed to the heterogeneous population, potential off‐target effects at these high doses, as well as baseline expression of K_v_1.4 (I_to_).

Using nifedipine, we demonstrated the functional differences in Ca^2+^ handling dynamics between hiPSC‐aCMs and ‐vCMs. Nifedipine elicited a dose‐dependent response in hiPSC‐vCMs demonstrating high sensitivity at 300 nM thereby confirming the functional presence of Ca_v_1.2. In contrast, hiPSC‐aCMs were relatively insensitive to nifedipine showing no statistically significant differences in APD at all tested doses. This finding is further corroborated by the relatively decreased expression of *CACNA1C* (Ca_v_1.2) in the hiPSC‐aCMs. This suggests that Ca^2+^ handling in hiPSC‐aCMs may be reliant on other voltage‐gated Ca^2+^ channels such as Ca_v_1.3, as this Ca^2+^ channel is blocked less potently by nifedipine.^34^ Moreover, our qPCR assay confirmed that hiPSC‐aCMs had higher expression of *CACNA1D* (Ca_v_1.3).

AVE0118 is an experimental K^+^ channel blocker (I_to_, I_Kur_, and I_Kr_) that was predicted to demonstrate targeted effects in hiPSC‐aCMs. However, only a nuanced atrial specificity was observed in our assay. Although the effects were proportionally larger in hiPSC‐aCMs, AVE0118 prolonged early repolarization of both hiPSC‐aCMs and ‐vCMs in a similar fashion. The drug prolonged mid‐ and late‐repolarization at a lower dose (1 μM) in hiPSC‐aCMs showing minimal atrial specific effects. Interestingly, AVE0118 greatly affected Ca^2+^ handling in hiPSC‐aCMs compared with hiPSC‐vCMs with larger proportional prolongation of CaTD_50_ at all doses. These results were unexpected as AVE0118 is thought to be highly specific to hiPSC‐aCMs due to its I_Kur_ blocking component. Perhaps the observed mixed‐effects in both cell types is due to the drug binding to I_to_ (IC_50_: 3.4 μM) and I_Kr_ (IC_50_: 9.6 μM)^35^ which prolongs APD at the tested doses of 3 and 10 μM as genes encoding the channels producing the I_to_ (*KCNA4*) and I_Kr_ (*KCNH2*) were expressed in our hiPSC‐vCMs. The drug was also shown to be effective in terminating certain ventricular arrhythmias^36^ which was predicted based on our results of prolongation in the APD of hiPSC‐vCMs.

Next, we used UCL1684, a highly specific SK channel pore blocker, to assess the presence of functional SK channels in hiPSC‐aCMs. The SK channel has three paralogs but the SK3 channel variant (*KCNN3*) has been shown to be atrial‐specific and has been implicated in AF pathogenesis in several studies.^37^, ^38^ In this study, UCL1684 displayed high specificity toward hiPSC‐aCMs with a strong dose‐dependent response. The drug confirmed the presence of functional SK channels in hiPSC‐aCMs at 3 μM with a positive dose‐dependent response while having no effect on hiPSC‐vCMs at all tested doses (0.3, 1, 3, and 10 μM).

Vernakalant is touted as an atrial‐selective compound clinically approved for intravenous cardioversion of AF.^39^ Strikingly, out of all the tested drugs, vernakalant showed the most pronounced atrial‐selective effects even though it is a blocker of multiple ion channels (I_Na_, I_Kur_, and I_K,Ach_). Vernakalant prolonged APD and CaTD of hiPSC‐aCMs at three tested doses (3, 10, and 30 μM). However, no statistically significant changes were observed in hiPSC‐vCMs at early‐ and mid‐ repolarization while the slight prolongation at APD_80_ at the clinically relevant dose (30 μM) may be attributed to the I_Na_ blocking component of vernakalant. This result further demonstrates the sensitivity of the assay in establishing atrial‐selective drug effects.

This study has several limitations. One limitation in our findings is that we cannot directly compare the results from qPCR and NanoString as both assays have fundamental differences in technical principles and statistical methodologies. Taken together, however, both assays show the global changes in cell type specific gene markers and further validate the role of retinoic acid in directing the cardiac differentiation process toward an atrial lineage. The main limitation in this field is the maturation state of the hiPSC‐CMs as they have an overall immature phenotype with some crucial differences compared with adult cardiomyocytes.^40^ Nonetheless, we were able to observe the stark differences in genetic, protein, as well as functional signatures of AP and CaT in the two generated chamber‐specific cell types. Additionally, maturation stage does not explain the differences in chamber‐specific phenotype as parallel batch differentiation and time‐in‐culture were incorporated in our study design. Most importantly, we were able to capture effects of drugs that were expected to have atrial‐specific properties in hiPSC‐aCMs.

## 5 CONCLUSION

The ability to differentiate hiPSC‐aCMs provides a unique opportunity to study atrial physiology and its pharmacologic responses in a human‐relevant in vitro model. We demonstrated an hiPSC‐based in vitro model that recapitulates the molecular and functional characteristics of the phenotype of native atrial tissue. Our platform adds to the repertoire of cardiac drug screening and can be readily applied in future efforts of atrial‐specific drug discovery.

## CONFLICT OF INTEREST

The authors declared no potential conflicts of interest.

## AUTHOR CONTRIBUTIONS

M.G.G., S.S.S.: conception and design, collection and assembly of data, data analysis and interpretation, manuscript writing; S.S.: experimental design support, data interpretation, manuscript writing; E.L.: designed and built the optical mapping system (hardware and software); D.A.H.‐W.: cell culture; V.J.B.: data collection, data analysis and interpretation, manuscript writing; Z.L., G.F.T.: conception of study, manuscript writing support and review, data interpretation, financial support.

## Supporting information


**Data S1**: Supporting Information

## Data Availability

The data that support the findings of this study are available on request from the corresponding author.

## References

[1] Karakikes I , Ameen M , Termglinchan V , Wu JC . Human induced pluripotent stem cell‐derived cardiomyocytes: insights into molecular, cellular, and functional phenotypes. Circ Res. 2015;117(1):80‐88. 10.1161/CIRCRESAHA.117.305365. 26089365 PMC4546707

[2] Xavier‐Neto J , Neville C . A retinoic acid‐inducible transgenic marker of sino‐atrial development in the mouse heart. Development. 1999;2687:2677‐2687. 10.1242/dev.126.12.2677 10331979

[3] Niederreither K , Vermot J , Schuhbaur B , Chambon P , Dollé P . Embryonic retinoic acid synthesis is essential for heart morphogenesis in the mouse. Development. 2001;128:1019‐1031. 11245568 10.1242/dev.128.7.1019

[4] Hochgreb T , Linhares VL , Menezes DC , et al. A caudorostral wave of RALDH2 conveys anteroposterior information to the cardiac field. Development. 2003;130(22):5363‐5374. 10.1242/dev.00750. 13129847

[5] Moss JB , Xavier‐Neto J , Shapiro MD , et al. Dynamic patterns of retinoic acid synthesis and response in the developing mammalian heart. Dev Biol. 1998;199(1):55‐71. 10.1006/dbio.1998.8911. 9676192

[6] Lee JH , Protze SI , Laksman Z , Backx PH , Keller GM . Human pluripotent stem cell‐derived atrial and ventricular cardiomyocytes develop from distinct mesoderm populations. Cell Stem Cell. 2017;21(2):179‐194.e4. 10.1016/j.stem.2017.07.003. 28777944

[7] Laksman Z , Wauchop M , Lin E , et al. Modeling atrial fibrillation using human embryonic stem cell‐derived atrial tissue. Sci Rep. 2017;7:5268 10.1038/s41598-017-05652-y. 28706272 PMC5509676

[8] Devalla HD , Schwach V , Ford JW , et al. Atrial‐like cardiomyocytes from human pluripotent stem cells are a robust preclinical model for assessing atrial‐selective pharmacology. EMBO Mol Med. 2015;7(4):394‐410. 10.15252/emmm.201404757. 25700171 PMC4403042

[9] Argenziano M , Lambers E , Hong L , et al. Electrophysiologic characterization of calcium handling in human induced pluripotent stem cell‐derived atrial Cardiomyocytes. Stem Cell Rep. 2018;10(6):1867‐1878. 10.1016/j.stemcr.2018.04.005. PMC5989733 29731429

[10] Cyganek L , Tiburcy M , Sekeres K , et al. Deep phenotyping of human induced pluripotent stem cell‐derived atrial and ventricular cardiomyocytes. JCI Insight. 2018;3(12):1–17. 10.1172/jci.insight.99941. PMC6124434 29925689

[11] Lian X , Zhang J , Azarin SM , et al. Directed cardiomyocyte differentiation from human pluripotent stem cells by modulating Wnt/β‐catenin signaling under fully defined conditions. Nat Protoc. 2013;8:162‐175. 10.1038/nprot.2012.150. 23257984 PMC3612968

[12] Shafaattalab S , Li AY , Lin E , et al. In vitro analyses of suspected arrhythmogenic thin filament variants as a cause of sudden cardiac death in infants. Proc Natl Acad Sci USA. 2019;201819023:6969‐6974. 10.1073/pnas.1819023116. PMC6452669 30886088

[13] Shafaattalab S , Lin E , Christidi E , et al. Ibrutinib displays atrial‐specific toxicity in human stem cell‐derived cardiomyocytes. Stem Cell Rep. 2019;12(5):996‐1006. 10.1016/j.stemcr.2019.03.011. PMC6524928 31031187

[14] Pei F , Jiang J , Bai S , et al. Chemical‐defined and albumin‐free generation of human atrial and ventricular myocytes from human pluripotent stem cells. Stem Cell Res. 2017;19:94‐103. 10.1016/j.scr.2017.01.006. 28110125

[15] Zhang Q , Jiang J , Han P , et al. Direct differentiation of atrial and ventricular myocytes from human embryonic stem cells by alternating retinoid signals. Cell Res. 2011;21(4):579‐587. 10.1038/cr.2010.163. 21102549 PMC3203651

[16] Geiss GK , Bumgarner RE , Birditt B , et al. Direct multiplexed measurement of gene expression with color‐coded probe pairs. Nat Biotechnol. 2008;26(3):317‐325. 10.1038/nbt1385. 18278033

[17] Roden D , Cardiovascular Drugs WJT . Cardiovascular Drugs. Circulation. 2012;102(5):415‐415. 10.1161/01.cir.97.5.415.

[18] Ravens U . Atrial‐selective K^+^ channel blockers: potential antiarrhythmic drugs in atrial fibrillation? Can J Physiol Pharmacol. 2017;95(11):1313‐1318. 10.1139/cjpp-2017-0024. 28738160

[19] Wang Y , Tang S , Harvey KE , et al. Molecular determinants of the differential modulation of Ca_v_1.2 and Ca_v_1.3 by nifedipine and FPL 64176 s. Mol Pharmacol. 2018;94:973‐983. 10.1124/mol.118.112441. 29980657 PMC11033928

[20] Wettwer E , Hála O , Christ T , et al. Role of I_Kur_ in controlling action potential shape and contractility in the human atrium: influence of chronic atrial fibrillation. Circulation. 2004;110(16):2299‐2306. 10.1161/01.CIR.0000145155.60288.71. 15477405

[21] Strøbaek D , Jørgensen TD , Christophersen P , Ahring PK , Olesen SP . Pharmacological characterization of small‐conductance Ca^2+^‐activated K^+^ channels stably expressed in HEK 293 cells. Br J Pharmacol. 2000;129(5):991‐999. 10.1038/sj.bjp.0703120. 10696100 PMC1571906

[22] Finnin M . Vernakalant: a novel agent for the termination of atrial fibrillation. Am J Heal Pharm. 2010;67(14):1157‐1164. 10.2146/ajhp080501. 20592320

[23] Roy D , Pratt CM , Torp‐Pedersen C , et al. Vernakalant hydrochloride for rapid conversion of atrial fibrillation: a phase 3, randomized, placebo‐controlled trial. Circulation. 2008;117(12):1518‐1525. 10.1161/CIRCULATIONAHA.107.723866. 18332267

[24] Jonsson M , Synnergren J , Jeppsson A , Dellgren G , Asp J . Comparison of human cardiac gene expression profiles in paired samples of right atrium and left ventricle collected in vivo. Physiol Genomics. 2011;44(1):89‐98. 10.1152/physiolgenomics.00137.2011. 22085905

[25] Niwa N , Nerbonne JM . Molecular determinants of cardiac transient outward potassium current (Ito) expression and regulation. J Mol Cell Cardiol. 2010;48(1):12‐25. 10.1016/j.yjmcc.2009.07.013. 19619557 PMC2813406

[26] The Genotype‐Tissue Expression (GTEx) Project was supported by the Common Fund of the Office of the Director of the National Institutes of Health, and by NCI, NHGRI, NHLBI, NIDA, NIMH, and NINDS. The data used for the analyses described in this manuscript.

[27] Burridge PW , Matsa E , Shukla P , et al. Chemically defned generation of human cardiomyocytes. Nat Methods. 2014;11(8):855‐860. 10.1038/nMeth.2999. 24930130 PMC4169698

[28] Brandenburg S , Kohl T , Williams GSB , et al. Axial tubule junctions control rapid calcium signaling in atria. J Clin Invest. 2016;126(10):3999‐4015. 10.1172/JCI88241. 27643434 PMC5096811

[29] Goldhaber JI , Xie L‐H , Duong T , Motter C , Khuu K , Weiss JN . Action potential duration restitution and alternans in rabbit ventricular myocytes. Circ Res. 2005;96(4):459‐466. 10.1161/01.res.0000156891.66893.83. 15662034

[30] Shattock MJ , Park KC , Yang H‐Y , et al. Restitution slope is principally determined by steady‐state action potential duration. Cardiovasc Res. 2017;113(7):817‐828. 10.1093/cvr/cvx063. 28371805 PMC5437364

[31] Lip GYH , Fauchier L , Freedman SB , et al. Atrial fibrillation. Nat Rev Dis Prim. 2016;2:16016 10.1038/nrdp.2016.16. 27159789

[32] Jaiswal A , Goldbarg S . Dofetilide induced torsade de pointes: mechanism, risk factors and management strategies. Indian Heart J. 2014;66:640‐648. 10.1016/j.ihj.2013.12.021. 25634399 PMC4311014

[33] Wang Z , Fermini B , Nattel S . Effects of flecainide, quinidine, and 4‐aminopyridine on transient outward and ultrarapid delayed rectifier currents in human atrial myocytes. J Pharmacol Exp Ther. 1995;272(1):184‐196. 7815332

[34] Wang Y , Tang S , Harvey KE , et al. Molecular determinants of the differential modulation of Ca_v_1.2 and Ca_v_1.3 by nifedipine and FPL 64176. Mol Pharmacol. 2018;94(3):973‐983. 10.1124/mol.118.112441. 29980657 PMC11033928

[35] Gogelein H , Brendel J , Steinmeyer K , et al. Effects of the atrial antiarrhythmic drug AVE0118 on cardiac ion channels. Naunyn Schmiedebergs Arch Pharmacol. 2004;370(3):183‐192. 10.1007/s00210-004-0957-y. 15340774

[36] Billman GE , Kukielka M . Novel transient outward and ultra‐rapid delayed rectifier current antagonist, AVE0118, protects against ventricular fibrillation induced by myocardial ischemia. J Cardiovasc Pharmacol. 2008;51(4):352‐358. 10.1097/FJC.0b013e31816586bd. 18427277

[37] Diness JG , Sørensen US , Nissen JD , et al. Inhibition of small‐conductance Ca^2+^‐activated K^+^ channels terminates and protects against atrial fibrillation. Circ Arrhythmia Electrophysiol. 2010;3(4):380‐390. 10.1161/CIRCEP.110.957407. 20562443

[38] Diness JG , Skibsbye L , Jespersen T , et al. Effects on atrial fibrillation in aged hypertensive rats by Ca^2+^‐activated K^+^ channel inhibition. Hypertension. 2011;57(6):1129‐1135. 10.1161/HYPERTENSIONAHA.111.170613. 21502564

[39] Canada H . Brinavess Product Monograph. Uxbridge: Cardiome; 2018:1‐33.

[40] Tu C , Chao BS , Wu JC . Strategies for improving the maturity of human induced pluripotent stem cell‐derived cardiomyocytes. Circ Res. 2018;123(5):512‐514. 10.1161/CIRCRESAHA.118.313472. 30355143 PMC6392006

