# Supplementary material for: Drug screening platform using human induced pluripotent stem cell‐derived atrial cardiomyocytes and optical mapping
Source: Stem Cells Transl Med. 2020 Sep 14;10(1):68–82. doi: 10.1002/sctm.19-0440 (PMC7780813; doi:10.1002/sctm.19-0440)
Supplement: Supplementary file 1 — Data S1: Supporting Information [file SCT3-10-68-s001.docx]

**Figure S1: Transcripts of hiPSC-aCMs and -vCMs assessed by the qPCR assay.**

qPCR analysis of various genes in atrial and ventricular cardiomyocytes including atrial markers S*LN*, and *TBX5*. The pan-cardiac marker *TNNT2*, as well as *SHOX2, HCN4*, and *MYL7*. n = 3 differentiation batches, unpaired t-test, *p<0.05, ***p<0.001.

**Figure S2: Optimization of retinoic acid (RA) dose and time window based on cardiogenesis efficiency and gene expression changes.**

A) Flow cytometric analysis of cTnT expression following the dose titration of retinoic acid (RA) with daily additions over days 3-6 (right panel) and days 4-6 (left panel). n = 4 independent differentiation batches, unpaired t-test comparing the dose of RA to vehicle control (DMSO), *p<0.05, **p<0.01, ***p<0.001. B) qPCR expression profile of cardiomyocytes with RA added from days 3-6 compared to the vehicle control. Atrial markers *NPPA* and *KCNA5* were shown to have significantly higher expression compared to the vehicle control. However, other atrial markers, *CACNA1D, SLN, KCNJ3, GJA5, TBX5* were not expressed at a significantly higher level compared to the vehicle control. n = 3 independent differentiation batches, unpaired t-test, *p<0.05.

**Figure S3: Optimization of retinoic acid (RA) addition timeframe as measured by dual fluorochrome flow cytometry.**

Flow cytometric analysis of cTnT (pan-cardiac marker) and MLC-2v (ventricular marker) expression after the addition of 0.75 µM retinoic acid or DMSO (vehicle control) from days 3-6 or 4-6. Upper panel contains representative contour plots of cTnT (x-axis) and MLC-2V expression (y-axis). Bottom panel presents the quantification of cTnT (orange bars) as proportion of the total population and MLC-2v (blue bars) expression as proportion of cTnT-positive population. n = 4 independent differentiation batches, unpaired t-test, *p<0.05, **p<0.01, ***p<0.001.


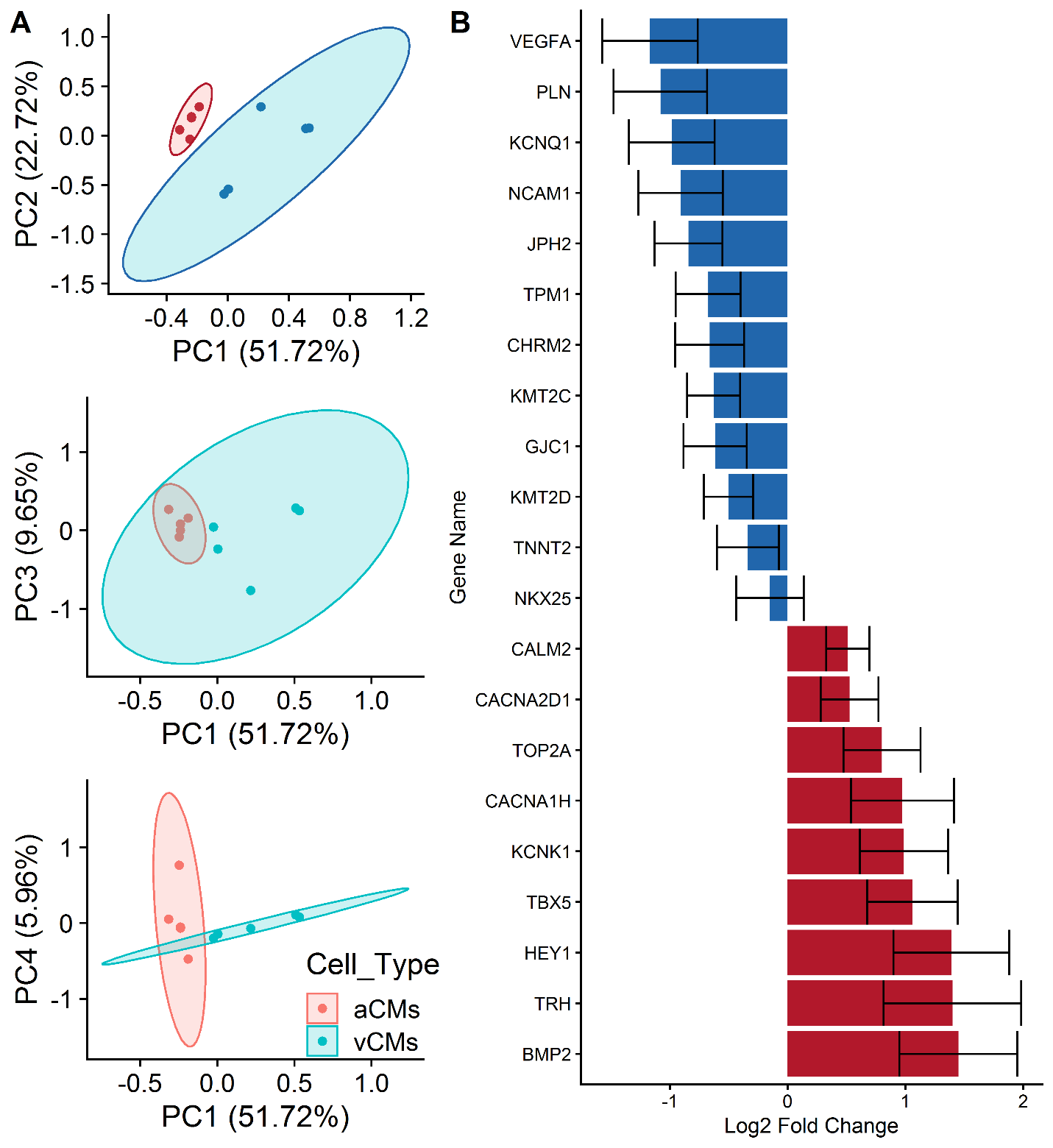


**Figure S4: Extended analysis of the NanoString assay.**

A) Principal component analysis (PCA) illustrating the first principal component plotted against the second principal component (top panel), the third principal component (middle panel) and the fourth principal component (bottom panel). B) Selected list of transcripts of the NanoString assay that were found to have no statistically significant differences based on the FDR correction.

**Figure S5: Cardiac enrichment using magnetic- activated cell sorting (MACS).**

Flow cytometry analyses of pre-enriched and post-enriched population of hiPSC-CMs. A) Histogram showing the distribution of cells expressing cardiac troponin T (cTnT) in unstained, pre-enriched, and post-enriched HiPSC-CMs. The blue box indicates the gating that measures the fraction of cTnT positive cells. B) Quantification of cTnT expressing population of pre- and post-enriched hiPSC-CMs, unpaired t-test, *p < 0.05.


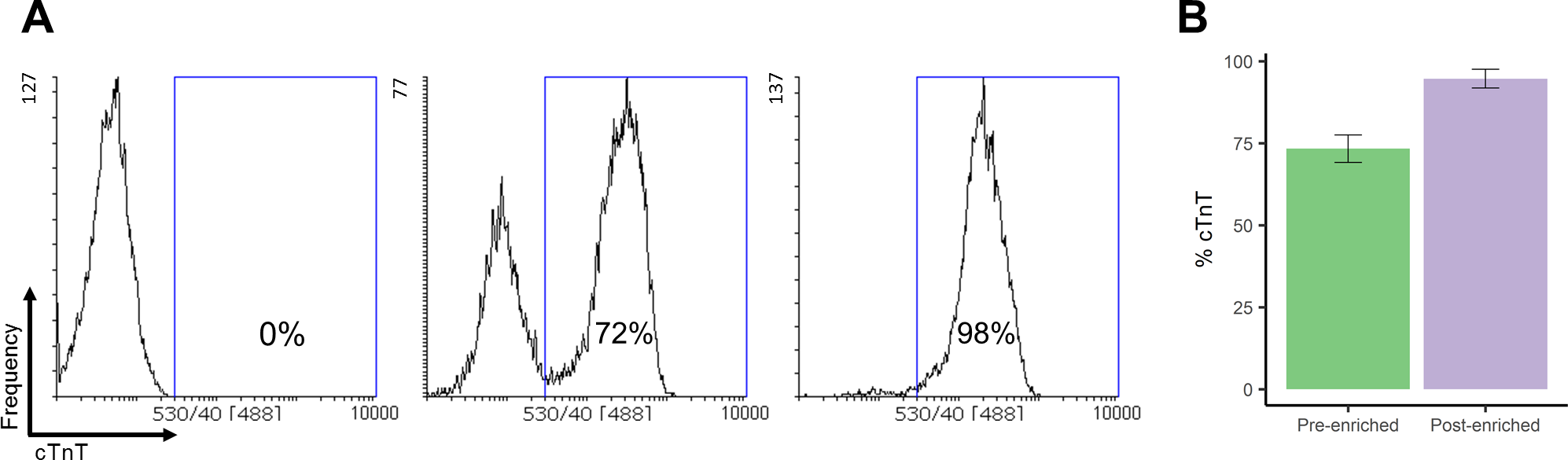


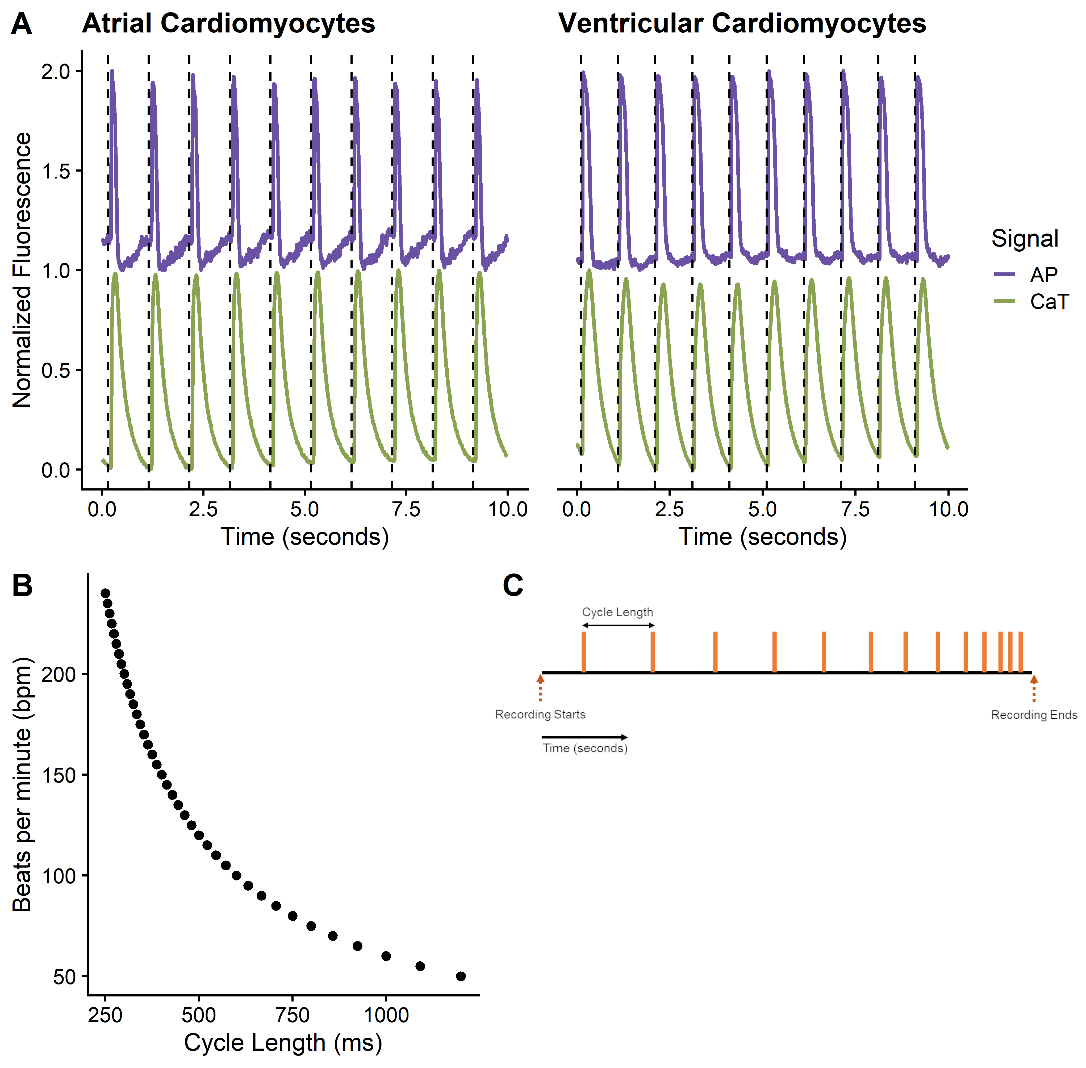


**Figure S6: Pacing protocol and rate dependence of hiPSC-aCMs and –vCMs.**

A) Representative traces of action potential (purple) and Ca^2+^ transient (green) of hiPSC-aCMs and -vCMs paced at 1 Hz. Pacing events are denoted by the dashed line. B) Relationship between beating rate (beats per minute: BPM) and cycle length (CL). CL was used to measure the diastolic interval (DI = CL - APD_80_). C) An example of the variable rate pacing protocol used for investigating rate-dependent properties of the cardiac tissues. Orange bars denote pulse width stimulation of 5V in amplitude and 5 msec in duration.

**
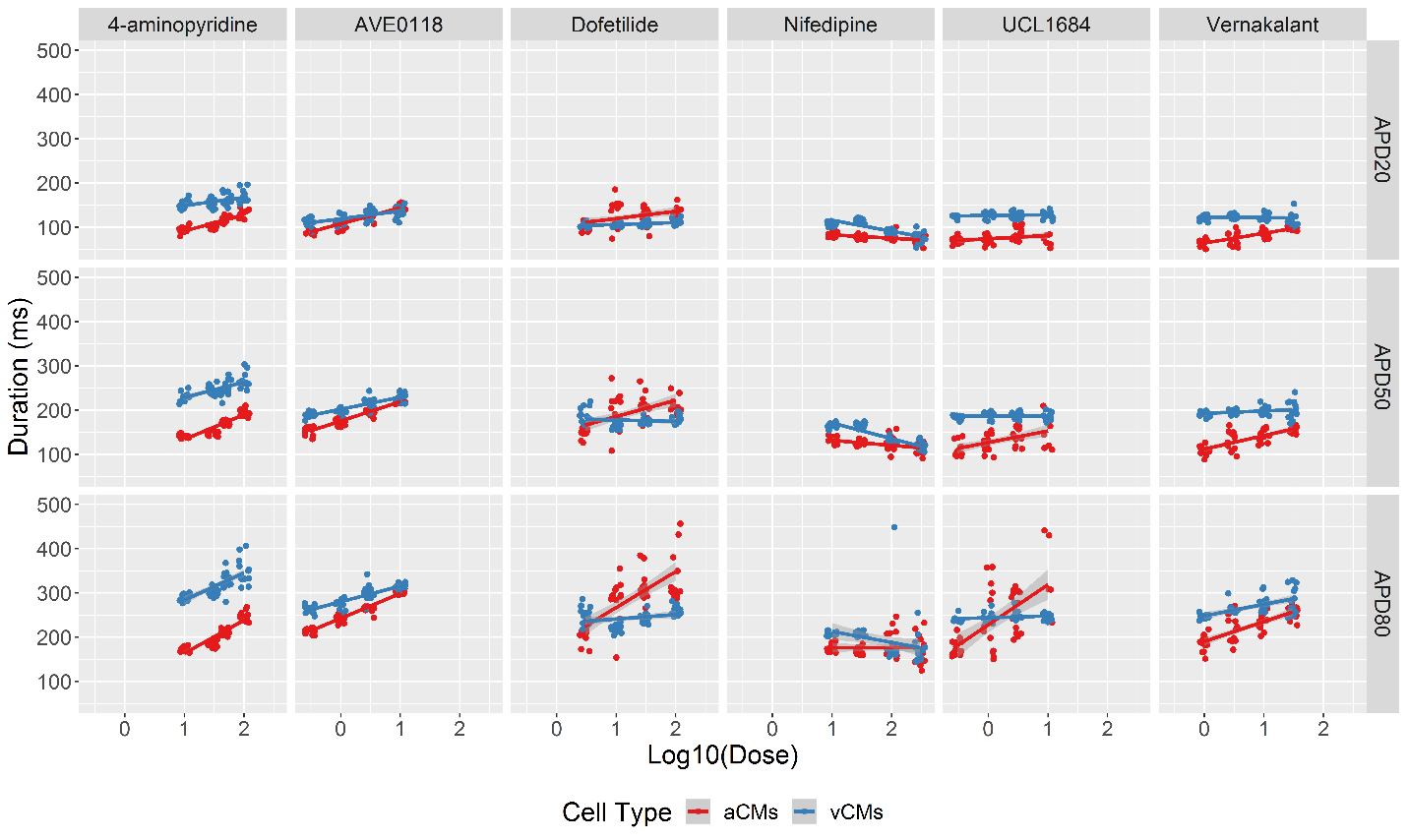
**

**Figure S7: Linear model representation to illustrate the comparison of dose-dependent drug effects on the action potential duration (APD) of hiPSC-aCMs and -vCMs.**

A linear model was fitted on data points obtained from sampling 13 regions of interest (ROIs) across 6 independent experiments (n = 6 from six differentiation batches). The dose units for dofetilide and nifedipine are expressed in nM while the units for the other compounds are expressed in µM. hiPSC-aCMs (red) and -vCMs (blue).

**
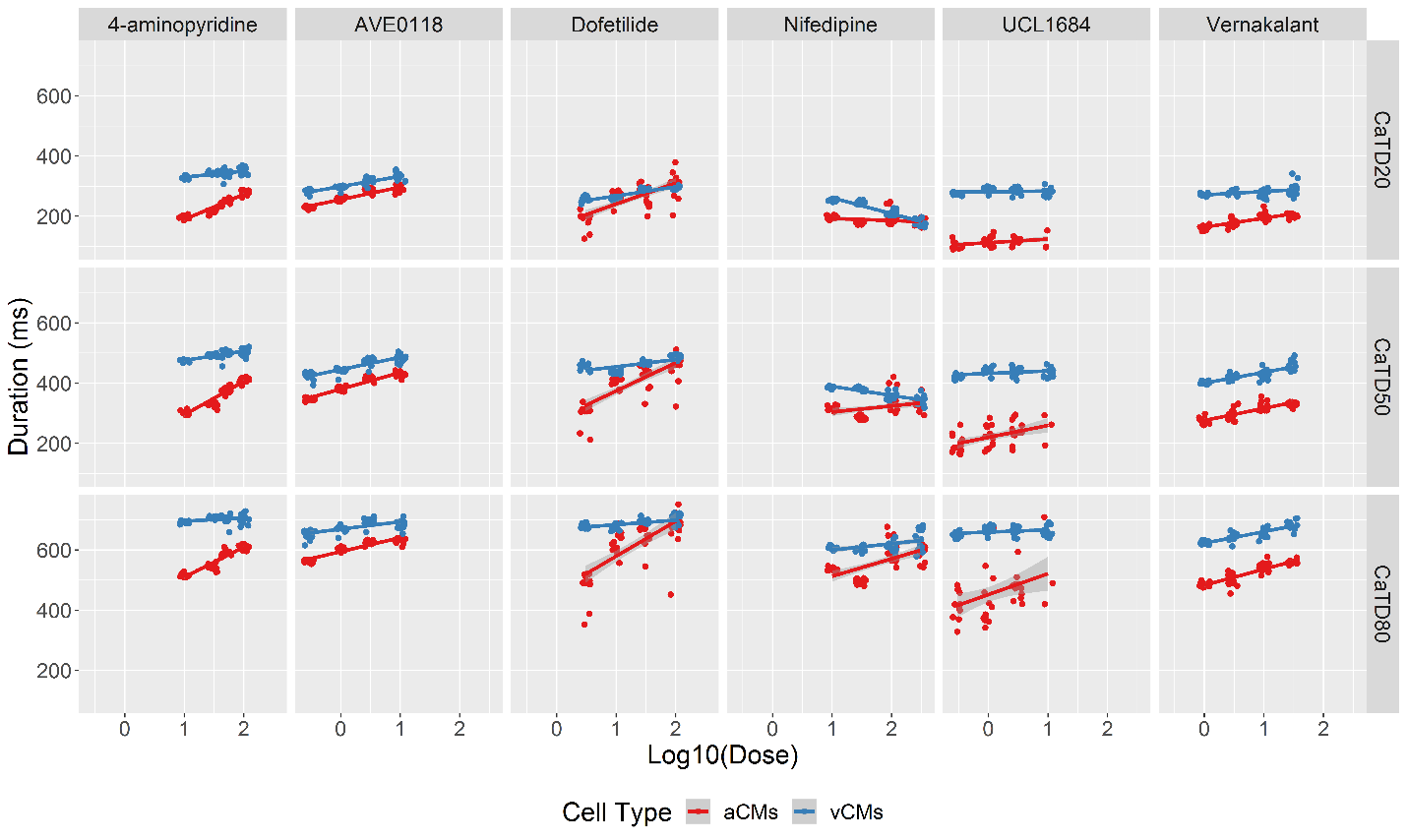
**

**Figure S8: Linear model representation to illustrate the comparison of dose-dependent drug effects on calcium transient duration (CaTD) of hiPSC-aCMs and -vCMs.**

A linear model was fitted on data points obtained from sampling 13 regions of interest (ROIs) across 6 independent experiments (n = 6 from 6 differentiation batches). The dose unit for dofetilide and nifedipine are expressed in nM while the units for the other compounds are expressed in µM. hiPSC-aCMs (red) and -vCMs (blue).

**Table S1: The effects of dofetilide on hiPSC-derived atrial and ventricular CMs.** Measured parameters are early-, mid-, and late- repolarization (APD_20_, APD_50_, and APD_80_) and Ca^2+^ decay (CaTD_20_, CaTD_50_, and CaTD_80_). Data are presented as mean ± SEM with unit expressed in milliseconds. Highlighted cells in orange represent statistically significant change (p < 0.05) compared to control as determined by one-way ANOVA and Dunnett’s post-hoc test. n = 6 independent differentiation batches.

| Dofetilide (nM) | 0 | 3 | 10 | 30 | 100 |
| --- | --- | --- | --- | --- | --- |
| **Atrial CMs** |  |  |  |  |  |
| APD20 | 63 ± 3 | 61 ± 4 | 75 ± 5 | 63 ± 4 | 68 ± 4 |
| APD50 | 98 ± 4 | 96 ± 4 | 122 ± 6 | 131 ± 12 | 129 ± 8 |
| APD80 | 182 ± 26 | 241 ± 26 | 279 ± 15 | 350 ± 33 | 355 ± 24 |
| CaTD20 | 117 ± 2 | 128 ± 2 | 147 ± 4 | 165 ± 4 | 185 ± 5 |
| CaTD50 | 178 ± 3 | 205 ± 5 | 226 ± 6 | 260 ± 6 | 289 ± 5 |
| CaTD80 | 311 ± 7 | 384 ± 13 | 419 ± 12 | 491 ± 12 | 467 ± 7 |
| **Ventricular CMs** |  |  |  |  |  |
| APD20 | 105 ± 6 | 120 ± 3 | 151 ± 16 | 140 ± 17 | 127 ± 13 |
| APD50 | 162 ± 4 | 187 ± 5 | 228 ± 19 | 222 ± 22 | 202 ± 18 |
| APD80 | 238 ± 20 | 262 ± 9 | 316 ± 21 | 310 ± 28 | 319 ± 45 |
| CaTD20 | 228 ± 6 | 259 ± 7 | 307 ± 21 | 304 ± 26 | 288 ± 32 |
| CaTD50 | 346 ± 7 | 401 ± 6 | 461 ± 16 | 449 ± 28 | 443 ± 34 |
| CaTD80 | 599 ± 33 | 635 ± 12 | 674 ± 10 | 672 ± 37 | 662 ± 57 |

**Table S2: The effects of nifedipine on hiPSC-derived atrial and ventricular CMs.** Measured parameters are early-, mid-, and late- repolarization (APD_20_, APD_50_, and APD_80_) and Ca^2+^ decay (CaTD_20_, CaTD_50_, and CaTD_80_). Data are presented as mean ± SEM with unit expressed in milliseconds. Highlighted cells in orange represent statistically significant change (p < 0.05) compared to control as determined by one-way ANOVA and Dunnett’s post-hoc test. n = 6 independent differentiation batches.

| Nifedipine (nM) | 0 | 10 | 30 | 100 | 300 |
| --- | --- | --- | --- | --- | --- |
| **Atrial CMs** |  |  |  |  |  |
| APD20 | 73 ± 3 | 81 ± 4 | 74 ± 5 | 71 ± 8 | 70 ± 5 |
| APD50 | 116 ± 5 | 130 ± 5 | 120 ± 7 | 125 ± 14 | 112 ± 7 |
| APD80 | 154 ± 7 | 174 ± 7 | 166 ± 7 | 182 ± 27 | 160 ± 18 |
| CaTD20 | 172 ± 11 | 192 ± 10 | 170 ± 13 | 186 ± 18 | 171 ± 9 |
| CaTD50 | 265 ± 19 | 302 ± 18 | 277 ± 13 | 327 ± 31 | 310 ± 14 |
| CaTD80 | 438 ± 36 | 497 ± 34 | 466 ± 17 | 531 ± 42 | 552 ± 39 |
| **Ventricular CMs** |  |  |  |  |  |
| APD20 | 114 ± 13 | 108 ± 12 | 116 ± 13 | 90 ± 11 | 70 ± 10 |
| APD50 | 170 ± 14 | 165 ± 15 | 166 ± 14 | 130 ± 14 | 121 ± 16 |
| APD80 | 214 ± 17 | 208 ± 17 | 208 ± 16 | 168 ± 17 | 177 ± 22 |
| CaTD20 | 240 ± 9 | 254 ± 10 | 247 ± 7 | 209 ± 8 | 169 ± 18 |
| CaTD50 | 357 ± 10 | 386 ± 10 | 381 ± 10 | 357 ± 10 | 333 ± 23 |
| CaTD80 | 570 ± 15 | 608 ± 12 | 603 ± 16 | 612 ± 17 | 627 ± 43 |

**Table S3: The effects of 4-aminopyridine on hiPSC-derived atrial and ventricular CMs.** Measured parameters are early-, mid-, and late- repolarization (APD_20_, APD_50_, and APD_80_) and Ca^2+^ decay (CaTD_20_, CaTD_50_, and CaTD_80_). Data are presented as mean ± SEM with unit expressed in milliseconds. Highlighted cells in orange represent statistically significant change (p < 0.05) compared to control as determined by one-way ANOVA and Dunnett’s post-hoc test. n = 6 independent differentiation batches.

| 4AP (µM) | 0 | 10 | 30 | 50 | 100 |
| --- | --- | --- | --- | --- | --- |
| **Atrial CMs** |  |  |  |  |  |
| APD20 | 82 ± 8 | 95 ± 10 | 101 ± 8 | 120 ± 9 | 131 ± 9 |
| APD50 | 118 ± 10 | 141 ± 12 | 148 ± 10 | 172 ± 11 | 197 ± 10 |
| APD80 | 149 ± 11 | 173 ± 14 | 184 ± 12 | 212 ± 13 | 248 ± 11 |
| CaTD20 | 157 ± 8 | 196 ±10 | 216 ± 9 | 250 ± 8 | 279 ± 8 |
| CaTD50 | 244 ± 11 | 303 ± 14 | 328 ± 14 | 379 ± 10 | 410 ± 10 |
| CaTD80 | 427 ± 21 | 516 ± 21 | 541 ± 20 | 591 ± 14 | 610 ± 10 |
| **Ventricular CMs** |  |  |  |  |  |
| APD20 | 138 ± 8 | 152 ± 8 | 152 ± 11 | 163 ± 13 | 169 ± 9 |
| APD50 | 209 ± 10 | 230 ± 10 | 244 ± 11 | 248 ± 16 | 267 ± 12 |
| APD80 | 281 ± 16 | 289 ± 10 | 303 ± 11 | 323 ± 17 | 345 ± 17 |
| CaTD20 | 302 ± 11 | 329 ± 8 | 344 ± 7 | 341 ± 14 | 350 ± 15 |
| CaTD50 | 451 ± 15 | 474 ± 10 | 493 ± 9 | 498 ± 14 | 504 ± 15 |
| CaTD80 | 678 ± 15 | 693 ± 10 | 708 ± 9 | 703 ± 15 | 705 ± 15 |

**Table S4: The effects of AVE0118 on hiPSC-derived atrial and ventricular CMs.** Measured parameters are early-, mid-, and late- repolarization (APD_20_, APD_50_, and APD_80_) and Ca^2+^ decay (CaTD_20_, CaTD_50_, and CaTD_80_). Data are presented as mean ± SEM with unit expressed in milliseconds. Highlighted cells in orange represent statistically significant change (p < 0.05) compared to control as determined by one-way ANOVA and Dunnett’s post-hoc test. N = 6 independent differentiation batches.

| AVE0118 (µM) | 0 | 0.3 | 1 | 3 | 10 |
| --- | --- | --- | --- | --- | --- |
| **Atrial CMs** |  |  |  |  |  |
| APD20 | 88 ± 9 | 92 ± 16 | 105 ± 13 | 123 ± 12 | 147 ± 13 |
| APD50 | 138 ± 9 | 154 ± 17 | 174 ± 13 | 195 ± 12 | 220 ± 11 |
| APD80 | 200 ± 14 | 214 ± 17 | 244 ± 16 | 265 ± 15 | 302 ± 15 |
| CaTD20 | 178 ± 12 | 230 ± 15 | 257 ± 13 | 285 ± 11 | 290 ± 8 |
| CaTD50 | 277 ± 13 | 346 ± 15 | 382 ± 12 | 417 ± 10 | 427 ± 7 |
| CaTD80 | 462 ± 14 | 562 ± 9 | 602 ± 11 | 626 ± 9 | 633 ± 12 |
| **Ventricular CMs** |  |  |  |  |  |
| APD20 | 102 ± 10 | 113 ± 9 | 117 ± 7 | 132 ± 13 | 147 ± 14 |
| APD50 | 174 ± 15 | 191 ± 13 | 198 ± 12 | 218 ± 13 | 237 ± 13 |
| APD80 | 251 ± 16 | 265 ± 12 | 275 ± 13 | 301 ± 15 | 325 ± 14 |
| CaTD20 | 249 ± 22 | 283 ± 22 | 297 ± 21 | 318 ± 20 | 327 ± 18 |
| CaTD50 | 382 ± 21 | 426 ± 20 | 442 ± 17 | 473 ± 17 | 499 ± 19 |
| CaTD80 | 595 ± 20 | 652 ± 17 | 671 ± 12 | 699 ± 10 | 793 ± 88 |

**Table S5: The effects of UCL1684 on hiPSC-derived atrial and ventricular CMs.**  Measured parameters are early-, mid-, and late- repolarization (APD_20_, APD_50_, and APD_80_) and Ca^2+^ decay (CaTD_20_, CaTD_50_, and CaTD_80_). Data are presented as mean ± SEM with unit expressed in milliseconds. Highlighted cells in orange represent statistically significant change (p < 0.05) compared to control as determined by one-way ANOVA and Dunnett’s post-hoc test. N = 6 independent differentiation batches.

| UCL1684 (µM) | 0 | 0.3 | 1 | 3 | 10 |
| --- | --- | --- | --- | --- | --- |
| **Atrial CMs** |  |  |  |  |  |
| APD_20_ | 45 ± 6 | 47 ± 10 | 51 ± 8 | 61 ± 9 | 61 ± 5 |
| APD_50_ | 86 ± 6 | 87 ± 12 | 101 ± 10 | 115 ± 12 | 119 ± 11 |
| APD_80_ | 136 ± 11 | 140 ± 17 | 175 ± 27 | 188 ± 25 | 206 ± 32 |
| CaTD_20_ | 128 ± 12 | 129 ± 11 | 156 ± 12 | 167 ± 15 | 194 ± 21 |
| CaTD_50_ | 188 ± 10 | 209 ± 10 | 238 ± 10 | 261 ± 13 | 282 ± 26 |
| CaTD_80_ | 300 ± 15 | 372 ± 23 | 387 ± 33 | 413 ± 24 | 416 ± 39 |
| **Ventricular CMs** |  |  |  |  |  |
| APD20 | 110 ± 7 | 125 ± 8 | 129 ± 12 | 128 ± 13 | 127 ± 12 |
| APD50 | 165 ± 7 | 185 ± 10 | 190 ± 13 | 187 ± 16 | 185 ± 14 |
| APD80 | 222 ± 11 | 239 ± 9 | 247 ± 13 | 248 ± 18 | 244 ± 15 |
| CaTD20 | 243 ± 9 | 275 ± 14 | 292 ± 20 | 283 ± 25 | 272 ± 28 |
| CaTD50 | 379 ± 15 | 423 ± 21 | 444 ± 26 | 436 ± 33 | 425 ± 40 |
| CaTD80 | 600 ± 22 | 653 ± 23 | 670 ± 23 | 659 ± 35 | 649 ± 47 |

**Table S6: The effects of vernakalant on hiPSC-derived atrial and ventricular CMs.** Measured parameters are early-, mid-, and late- repolarization (APD_20_, APD_50_, and APD_80_) and Ca^2+^ decay (CaTD_20_, CaTD_50_, and CaTD_80_). Data are presented as mean ± SEM with unit expressed in milliseconds. Highlighted cells in orange represent statistically significant change (p < 0.05) compared to control as determined by one-way ANOVA and Dunnett’s post-hoc test. n = 6 independent differentiation batches.

| Vernakalant (µM) | 0 | 1 | 3 | 10 | 30 |
| --- | --- | --- | --- | --- | --- |
| **Atrial CMs** |  |  |  |  |  |
| APD20 | 44 ± 6 | 46 ± 7 | 61 ± 8 | 81 ± 6 | 98 ± 2 |
| APD50 | 80 ± 13 | 84 ± 14 | 111 ± 16 | 136 ± 11 | 156 ± 3 |
| APD80 | 128 ± 13 | 154 ± 18 | 189 ± 25 | 226 ± 20 | 255 ± 10 |
| CaTD20 | 125 ± 12 | 143 ± 21 | 172 ± 25 | 198 ± 20 | 203 ± 4 |
| CaTD50 | 211 ± 11 | 243 ± 20 | 280 ± 26 | 317 ± 24 | 331 ± 6 |
| CaTD80 | 395 ± 12 | 451 ± 18 | 490 ± 24 | 534 ± 21 | 560 ± 9 |
| **Ventricular CMs** |  |  |  |  |  |
| APD20 | 131 ± 20 | 121 ± 17 | 125 ± 13 | 123 ± 12 | 118 ± 13 |
| APD50 | 195 ± 22 | 191 ± 16 | 197 ± 14 | 197 ± 17 | 199 ± 20 |
| APD80 | 238 ± 22 | 246 ± 17 | 263 ± 19 | 275 ± 25 | 289 ± 30 |
| CaTD20 | 262 ± 23 | 272 ± 13 | 278 ± 15 | 274 ± 21 | 273 ± 26 |
| CaTD50 | 391 ± 30 | 402 ± 17 | 420 ± 19 | 431 ± 35 | 450 ± 32 |
| CaTD80 | 615 ± 29 | 623 ± 15 | 644 ± 19 | 658 ± 22 | 672 ± 38 |

**Table S7: Oligonucleotides used in the qPCR assays**

| **Subtype** | **Gene** | **Protein** | **Primer sequence 5’🡪3’ (Fwd/Rev)** |
| --- | --- | --- | --- |
| Atrial | *NPPA* | Atrial Natriuretic peptide | ACAGGATTGGAGCCCAGAG |
|  |  |  | GGAGCCTCTTGCAGTCTGTC |
| Atrial | *KCNA5* | Potassium voltage-gated channel, shaker-related subfamily, member 5, K_v_1.5 | CGAGGATGAGGGCTTCATTA |
|  |  |  | CTGAACTCAGGCAGGGTCTC |
| Atrial | *KCNJ3* | Potassium inwardly-rectifying channel, subfamily J, member 3, K_ir_3.1 | CTGCTCAAAGGATGACTTGT |
|  |  |  | CATGGAACTGGGAGTAATCA |
| Atrial | *KCNN2* | Small conductance calcium-activated potassium channel 2, SK2 | TAAGCCAGACCATCAGGCAG |
|  |  |  | GGGACCGCTCAGCATTGTAA |
| Atrial | *PITX2* | Paired-like homeodomain transcription factor 2 | GCTTGCGAGCAAGGGAGTGTA |
|  |  |  | CATTGCATCCACCAGAGAAACTATTC |
| Atrial | *CACNA1D* | Calcium channel, voltage-dependent, L type, alpha 1D subunit | GATGCGATAGGATGGGAATG |
|  |  |  | CCACTAAGGACACCAAGAAC |
| Unknown | *KCNN3* | Small conductance calcium-activated potassium channel 3, SK3 | CCTGTATGAGTCAGCCTTTC |
|  |  |  | AGCTCTAGGGACTTCTAACC |
| Nodal | *TBX3* | T-box transcription factor TBX3 protein | CTTGTGATGTTTTCAGAGCC |
|  |  |  | TTCTCTCTAAAAGCAAGCGT |
| Nodal | *HCN4* | Potassium/sodium hyperpolarization-activated cyclic nucleotide-gated channel 4 | GGAGTACCCCATGATGCGAA |
|  |  |  | CTTCTTGCCAATGCGGTCCA |
| Atrial | *GJA5* | Gap junction alpha-5 protein, connexin 40 | AATCTTCCTGACCACCCTGCATGT |
|  |  |  | CAGCCACAGCCAGCATAAAGACAA |
| Atrial | *NR2F2* | Chicken ovalbumin upstream promoter transcription factor 2 | TCACCCGCCAAACTAAAGGA |
|  |  |  | CTCTGCACCGCAAAACCATA |
| Atrial | *SLN* | Sarcolipin | GCTCAAGTTGGAGACAGCGAG |
|  |  |  | GGCTTCTCCTCACCTCCTGAAG |
| Nodal | *SHOX2* | Short stature homeobox 2 | TAAAGGTGTTCTCATAGGGGC |
|  |  |  | CCTGAACCTGCTGAAATGGC |
| Developmental: ANP activator | *TBX5* | T-box transcription factor 5 TBX5 protein | TACCACCACACCCATCAAC |
|  |  |  | ACACCAAGACAGGGACAGAC |
| Developmental: SA node specification | *TBX18* | T-box transcription factor 18 TBX18 protein | ACTGTCTTCACAACCGTCAC |
|  |  |  | CTTCCAAACCCATTCTGTTGC |
| Developmental: ANP repressor | *TBX20* | T-box transcription factor 20 TBX20 protein | CGAGGGTCAGCCTTTACAAC |
|  |  |  | GTTGCTATGGATGCTGTGCTG |
| Developmental | *GATA2* | GATA binding protein 2 | GACTACAGCAGCGGACTCTT |
|  |  |  | CTTCTGAACAGGAACGAGCC |
| Developmental | *GATA4* | GATA binding protein 4 | CCCCAATCTGTAGATATGTTTG |
|  |  |  | TGCCGTTCATCTTGTGGTAG |
| Developmental | *GATA6* | GATA binding protein 6 | GGGCTCTACATAGGCGTCAG |
|  |  |  | AAGCAGACACGAGTGGAGTG |
| Cardiac | *TNNT2^*^* | Cardiac Troponin T | TTCACCAAAGATCTGCTCCT |
|  |  |  | TACTGGTGTGGAGTGGGTG |
| Cardiac | *NKX2.5^*^* | Homeobox protein Nkx-2.5 | CTCCCAACATGACCCTGAGT |
|  |  |  | GACGGCGAGATAGCAAAGG |
| Ventricular | *IRX4^*^* | Iroquois-class homeodomain protein IRX-4 | TTCCGTTCTGAAGCGTGGTC |
|  |  |  | TGAAGCAGGCAATTATTGGTGT |
| Ventricular | *MYL2^*^* | Myosin light chain ventricular isoform | ACAGGGATGGCTTCATTGAC |
|  |  |  | CCGCTCCCTTAAGTTTCTCC |
| Atrial and some Ventricular | *MYL7^*^* | Myosin light chain atrial isoform | GGCAAAGGGGTGGTGAAC |
|  |  |  | TTCTCGTCTCCATGGGTGAT |
| Housekeeping | *GAPDH* | Glyceraldehyde 3-phosphate dehydrogenase | CATGTTCCAATATGATTCCAC |
|  |  |  | AGTCTTCTGGGTGGCAGTGAT |
| Housekeeping | *ACTB* | β-actin | ATTGCCGACAGGATGCAGAA |
|  |  |  | GGGCCGGACTCGTCATACTC |

Table S8: List of drugs, the primary targets, and doses used in this study.

| **Compound** | **Primary Target(s)** | **Dose Range** |
| --- | --- | --- |
| Dofetilide | I_Kr_ | 3, 10, 30, 100 nM |
| Vernakalant | I_Na_, I_Kur_, I_KACh_ | 1, 3, 10, 30 µM |
| AVE0118 | I_Kur_, I_KACh_,I_to_ | 0.3, 1, 3, 10 µM |
| UCL1848 | I_SK_ | 0.3, 1, 3, 10 µM |
| 4-aminopyridine | I_Kur_ | 10, 30, 50, 100 µM |
| Nifedipine | I_Ca,L_ | 10, 30, 100, 300 nM |

Table S9: List of genes in the custom NanoString Codeset panel

| **Gene name** | **Gene name** | **Gene name** | **Gene name** | **Gene name** |
| --- | --- | --- | --- | --- |
| ABCC8 | CPT1B | ISL1 | NFATC4 | ROR2 |
| ACADM | CRHR2 | ISL2 | NKX2-5 | RPS13 |
| ACTA1 | CS | JAG1 | NNT | RYR2 |
| B actin | CTNNB1 | JAK1 | NODAL | SCN4A |
| ACTC1 | CXCR4 | JAK2 | NOTCH1 | SCN5A |
| ACTN2 | CYP26A1 | JAK3 | NOTCH2 | SGK1 |
| ADRB1 | CYP26B1 | JPH2 | NOTCH3 | SHOX2 |
| ADRB2 | DKK1 | KCNA4 | NOTCH4 | SIRPA |
| AKT | DYNC1I1 | KCNA5 | NPPA | SLC16A1 |
| ALDH1A1 | DYNLL2 | KCND3 | NPPB | SLC2A1 |
| ALDH1A2 | EMILIN2 | KCNH2 | NR2F1 | SLC2A4 |
| ALDH1A3 | ERBB2 | KCNJ2 | NR2F2 | SLC8A1 |
| CD13 | FGF17 | KCNJ3 | ORAI1 | SLN |
| JCTN (ASPH) | FGF19 | KCNJ5 | PDGFRA | SOX2 |
| ATP1A2 | FGF2 | KCNJ8 | PDK2 | SPCS1 |
| ATP1A3 | FGF4 | KCNK1 | PDK4 | STIM1 |
| ATP2A2 | FGF8 | KCNK3 | p110a | TACR1 |
| ATP5F1 | PKBP1B | KCNK5 | PITX2 | TACR3 |
| B2M | FLNA | KCNN1 | PKP2 | TBX1 |
| BMP10 | FLNB | KCNN2 | PLCB1 | TBX18 |
| BMP2 | FOXA1 | KCNN3 | PLCB2 | TBX2 |
| BMP4 | FOXF1 | KCNQ1 | PLCD1 | TBX20 |
| BRCA1 | FOXH1 | KDR | PLCD3 | TBX3 |
| BTAF1 | GAPDH | KlF20A | PLCE1 | TBX5 |
| BTK | GATA2 | KIT | PLCG1 | T |
| CACNA1C | GATA4 | KLF4 | PLCG2 | TEC |
| CACNA1D | GATA6 | KMT2A | PLN | CD90 |
| CACNA1G | GJA1 | KMT2B | POLR2A | TNNI1 |
| CACNA1H | GJA5 | KMT2C | POU3F4 | TNNI3 |
| CACNA2D1 | GJC1 | KMT2D | POU5F1 | TNNT2 exon 5 |
| CALM1 | GNAI1 | LDHA | PPIA | TNNT2 |
| CALM2 | GNAI2 | LEFTY1 | PPP1CA | TOP2A |
| CALM3 | GNAI3 | LIN28 | PPP2CA | TPM1 |
| CALR | GNAO1 | JNK3 | PPP3CB | TRDN |
| CaMK2A | GNAS | JNK1-alpha | PRKACA | TRH |
| CaMK2B | GREM2 | JNK2-alpha | PRKACB | TRPC1 |
| CaMK2D | GYPA | MEF2c | PRKACG | TRPC2 |
| CaMK2G | HAND1 | MESP1 | PRKAR1A | TRPC3 |
| CASQ2 | HAND2 | MIXL1 | PRKAR1B | TRPC4 |
| CAV3 | HAS2 | IP3R | PRKAR2A | TRPC5 |
| CER1 | HCN1 | MSX2 | PRKAR2B | TRPC6 |
| CHRM2 | HCN2 | MYBPC3 | PRKCA | TRPC7 |
| CKB | HCN4 | MYC | PRKCB | TSHR |
| CKM | HEY1 | MYH6 | PRKCG | SSEA5 |
| COL1A1 | HEY2 | MYH7 | PKG1 | TTN |
| COL2A1 | HIF1A | MYL2 | PTGS2 | VCAM-1 |
| COL3A1 | HOPX | MYL7 | RARA | VEGFA |
| COL4A2 | HRC | MYO1E | RARB | WNT3 |
| COL4A3 | IPO8 | NANOG | RARG | WNT3A |
| COL9A2 | IRX4 | NCAM1 | RBM20 | ZFP42 |

# Detailed Methods

## hiPSC Maintenance and Expansion

Commercially available hiPSCs (WiCell, IMR90-1) were maintained and expanded in feeder-free culture using 6-well plates coated with Matrigel (Corning; Cat: 354230) diluted with a solution containing 1 mg:12 mL DMEM/F12 and mTeSR1 medium (STEMCELL Technologies, Cat: 85850). Using Versene (EDTA) (Lonza, Cat: 17-711E), hiPSCs were passaged every 4 days or ~80-85% confluency at 1:15 ratio. Passaged hiPSCs were cultured with mTeSR1 supplemented with 10 µM Y27632, a ROCK inhibitor, (Biogems, Cat: 1293823) for the first 24 hours. mTeSR1 media was exchanged daily during cell culture maintenance.

## Directed Differentiation of hiPSCs into Atrial and Ventricular Subtypes

hiPSCs at 80% confluence were incubated in 1 mL Accutase® (Innovative Cell Technologies catalog #: AT104) for 8 mins at 37°C. Cells were spun at 200 RCF for 5 min, then re-suspended in 2 mL mTeSR (STEMCELL Technologies, catalog # 85850) + 1 µM Y-27632 (ROCK inhibitor, Biogems, catalog: 1293823) and re-plated at 87 500 cells/cm^2^. Once the cells reached >95% confluence, differentiation was initiated (day 0) according to the GiWi protocol using CHIR99021 (Biogems, catalog #: 2520691) in RPMI-1640 (Gibco™ catalog #: 11875-085) medium supplemented by B27 minus insulin (Thermo Scientific, catalog #: A1895601). At day 2, medium was replaced with RPMI-1640 with B27 minus insulin. At day 3 cells were incubated with inclusion of the WNT inhibitor IWP-4 (Biogems, catalog #:6861787). At day 5 the medium was replaced with RPMI-1640 with B27 minus insulin. Then finally at day 7, the medium was replaced with cardiomyocyte maintenance media (RPMI-1640 with B27 with insulin). Thereafter, cardiomyocyte maintenance medium was replaced every 4 days. For the atrial differentiation protocol, retinoic acid (Sigma-Aldrich, catalog #: R2625) was added every 24 hours at 0.75 µM from days 4-6 (total exposure of 3 days).

## Cardiomyocyte Dissociation

Beating CMs were washed 1x in PBS^-/-^ then incubated in 0.25% trypsin EDTA (HyClone Protease, General Electric catalog #: SH30042.01) for 6-8 minutes at 37°C or until the cells were visibly beginning to lift. The trypsin was inactivated with a 6x (relative to trypsin) volume of DMEM/F12 (Gibco, catalog #: 11330-032) and 10% fetal bovine serum. The harvested cells were filtered through a 70 μm filter (Miltenyi, catalog #: 130-110-916) to produce single cells.

## Flow Cytometry

hiPSC-aCMs and –vCMs were harvested as outlined above (Cardiomyocyte Dissociation). The filtered cells were centrifuged at 200 RCF and aspirated, then fixed in 4.1% PFA solution (BD Bioscience catalog #: 554655) for 25 min. The cells were then washed and permeabilized in Saponin/FBS (BD Bioscience, catalog #: 554723). Cells were subsequently incubated in primary mouse-cTnT (Thermo catalog #: MA5-12960, 1:2000) and rabbit-MLC2V (Abcam, catalog #: ab79935, 1:1000) antibodies overnight. The next day, cells were washed 3x and incubated in secondary goat anti mouse Alexa-488 (Thermo, catalog #: A-21121, 1:500) and goat anti rabbit Alexa-647 (Thermo Scientific, catalog #: A-21245, 1:2000) antibodies for 1 hour, respectively. Cells were then washed 3x and resuspended in PBS-/- for analysis. All analyses were performed on the BD JAZZ Fluorescence Activated Cell Sorter and the FlowJo software package. The proportional expression of MLC-2v in each cardiac population was defined as MLC-2v^+^/cTnT^+^

## mRNA Expression Profiling

Gene expression profiling was conducted using multiplexed NanoString assay and Quantitative Real-time PCR (qPCR) assay. Pooled total RNA, which was used in both assays, was extracted using the Qiagen RNeasy mini kit (catalog #: 74104).

The extracted RNA was subsequently reverse transcribed into cDNA using the Qiagen QuantiTect Reverse Transcription Kit (catalog #: 205311). The Quantitative Real-time PCR assay was performed on a Bio-Rad CFX96 Touch™ instrument with Bio-Rad SsoFast™ EvaGreen Supermix (catalog #: 1725202). Oligonucleotide sequences are available in Table S8 of the Supplementary Information.

The multiplexed mRNA profiling was conducted using a custom Codeset containing 250 gene probes synthesized by NanoString Technologies Inc. and the NanoString nCounter® SPRINT Profiler. A total of 50 ng of purified RNA per sample were hybridized overnight (16 h) to the custom capture and reporter probes. Hybridized samples were loaded into each channel of the nCounter® SPRINT cartridge. Raw mRNA counts were collected, and the results were normalized to 7 housekeeping genes (*PIK3CA, ATP5F1, IPO8, PPIA, SPCS1, AKT,* and *RPS13*). Analysis was performed on the nSolver analysis software and the Advanced Analysis module (NanoString Technologies Inc.).

## Atrial Natriuretic Peptide Measurement

The levels of atrial natriuretic peptide (ANP) of the differentiated cardiomyocytes of iPSCs were measured using a competitive enzyme-linked immunosorbent assay (ELISA) from a commercially available kit (Invitrogen, Cat # EIAANP). Cell culture media was not exchanged for 72 hours and was harvested on the day of the assay. The assay was conducted according to the manufacturer’s protocol and was measured using a spectrophotometric plate reader (Molecular Devices SpectraMax).

## Cardiomyocyte Enrichment

For cardiac enrichment, hiPSC-aCMs and hiPSC-vCMs at Day 20-30 post-differentiation were dissociated into single cells as outline above. The single cell CMs were enriched using MidiMACS LS Magnet Columns (Miltenyi, Cat: 130-042-301) and the PSC-derived Cardiomyocyte Isolation Kit (Miltenyi, Cat: 130-110-188) according to the manufacturer’s protocol. Enriched hiPSC-CMs were seeded on Matrigel-coated 24-well plates at a seeding density of 650,000 cells per well and cultured in cardiomyocyte maintenance media supplemented with 10 µM Y27632 for the first 24 hours. Maintenance medium was refreshed every 4 days.

## Electrophysiology – Patch clamp recordings

Single hiPSC-aCMs and -vCMs were plated on gelatin (0.1%) and Geltrex (1:10) at 30,000 cells per well. After 48 hours in culture, glass electrodes with an impedance of 2.5-4 MΩ were used to achieve the whole-cell configuration with single hiPSC-CMs. The seal integrity was monitored and only cells with gigaohm seals were used for further analysis. The internal recording solution (in mM): KCl 135, HEPES 10, CaCl_2_ 2, EGTA 5, MgCl_2_ 3, Na-ATP 3, Na-GTP 2, and Na_2_-phosphocreatine 2. The external solution (in mM): NaCl 140, MgCl_2_ 1, HEPES 10, CaCl_2_ 1.2, Glucose 10, and Sodium pyruvate 1. Current recordings were performed using the Axon Instruments 700B amplifier and digitized at 20,000 samples/s. All recordings were performed at 33-35C as maintained by an in-line heater and circulation pump. For pacing at 1Hz, gradually increasing amounts of current were injected with a 1ms pulse width until reliable action potentials (APs) were triggered.

## Optical Mapping

Optical mapping recordings were performed on enriched hiPSC-aCMs and -vCMs cultured in a 24-well plate format as outlined above at Day 45-60 post-differentiation. Thirty minutes prior to recording, cardiomyocyte culture medium was changed to Ca^2+^ Tyrode’s solution (in mM: 117 NaCl, 5.7 KCl, 4.4 NaHCO_3_, 1.5 NaH_2_PO_4_-H_2_O, 1.7 MgCl_2_, 10 Na-HEPES (C_8_H_18_N_2_O_4_S), 5 glucose, 5 creatine, 5 Na-Pyruvic acid, 1.8 CaCl_2_) and equilibrated in the incubator (37°C/5% CO_2_). Then, the plate was transferred to the multi-well optical mapping instrument in which the temperature of the chamber was maintained at 37°C. The hiPSC-CMs were sequentially loaded with 5 µM RH-237 (Thermo Scientific, Cat: S1109) for 50 minutes, followed by co-incubation of 15 µM blebbistatin (Toronto Research Chemical, Cat: B592500) and 5 µM Rhod-2AM mixture for 40 minutes (Thermo Scientific, Cat: R1244). Blebbistatin, a myosin ATPase inhibitor, was used to reduce movement artefact.

The multi-well optical mapping system was custom engineered in the lab. One well of the 24-well plate was illuminated with four 530 nm mounted LEDs (Thorlabs; Cat: M530L4) to simultaneously excite both RH-237 and Rhod-2AM. Images were acquired at a frame rate of 100 frames/second by a single scientific grade CMOS camera, Orca Flash 4.0 V2 (Hamamatsu; Cat: C11440-22CU), that is equipped with an optical splitter. A custom-engineered motorized imaging stage controlled the well-to-well image acquisition. A programmable stimulation system was used to pace the cells. This system comprises of a pair of stainless-steel electrodes placed 1 cm apart were used to deliver biphasic field stimulation pulses to electrically pace the hiPSC-CMs. Data collection, image processing, and initial data analysis were accomplished using a software custom-written in Interactive Data Language (Harris Geospatial Solutions).

## Pharmacological Analyses

The following drugs were purchased: vernakalant (Toronto Research Chemical, Cat: V128620), dofetilide (Tocris, Cat:3757), nifedipine (Sigma-Aldrich, Cat: N7634), 4-aminopyridine (Sigma-Aldrich, Cat: 275875), UCL1684 (Tocris, Cat: 1310), and AVE 0018 (Axon Medchem, Cat: 2243). All drug stocks except for 4-aminopyridine were made by dissolving in DMSO (Sigma Aldrich, Cat: 472301) to a final concentration of 10 mM. 4-aminopyridine was diluted in Ca^2+^ Tyrode’s solution to a final concentration of 10 mM. Drug stocks were further diluted in Ca^2+^ Tyrode’s solution prior to pharmacological testing with the final DMSO concentration in the experimental solution not exceeding 0.03% (v/v). Drug effects were studied in serum-free conditions (i.e. Ca^2+^ Tyrode’s and drug only) at four doses by increasing drug concentration in the same well with recordings at 20-minute time points.

## Data and Statistical Analysis

The differential gene expression analysis from NanoString data comparing the hiPSC-aCMs to –vCMs was corrected using the Benjamini-Hochberg false discovery rate (FDR) procedure. Unsupervised hierarchical clustering using the bottom-up agglomerative method was applied to find the most similar samples in the dataset based on the expression of each individual gene. The results from the hierarchical clustering produced the heatmap and cluster dendrogram tree. qPCR and ELISA data were analyzed using unpaired t-test to compare the effects of RA on cell differentiation.

For the analysis of patch clamp recordings, spontaneously firing APs and those measured from pacing at 1Hz were analyzed using the Neuromatic suite of analysis tools (Igor Pro). The maximal upstroke velocity was determined by calculating the dV/dS of at least 5 APs and averaging the maximums for each trace. To measure the resting membrane potential, we averaged the membrane voltage during a 5 second epoch without spontaneous activity one minute after break-in. We used standard methods to calculate the action potential duration (APD) at both 50% (APD_50_) and 90% (APD_90_) of the peak voltage. Durations were compared using Student’s t-test.

Optical waveform analysis was performed by correcting the signal photobleaching rate using a polynomial fit. Duration measurement starts at 50% of upstroke and ends with the indicated percentage (20%, 50%, and 80%) of repolarization (i.e. APD_20_, APD_50_, and APD_80_) or Ca^2+^ transient decay (i.e. CaTD_20_, CaTD_50_, and CaTD_80_). CaT time-to-peak was defined as the time course measured from the shoulder of the upstroke to the peak/maxima of the CaT signal. Time constant (τ) of CaT was defined as the time course measured by an exponential fit of the CaT decay portion.

All optical mapping data were obtained from thirteen regions of interest from each well covering the whole surface area in between the stimulation electrode (1 cm^2^). A 1 cm^2^ of a tissue sheet comprised of ~350,000 cardiomyocytes.

Optical mapping measurements of baseline conditions assessing the effect of RA compared to the control differentiation protocol were analyzed using an unpaired t-test. In electrical restitution analysis, data points obtained from the APD_80_ at each DI were fitted to a mono-phasic exponential association function and the data were best fit to a non-linear least square. To analyze drug effects, the initial analysis was conducted to assess the effects of drugs within each cell type and find the dose of significance. This was conducted using one-way ANOVA and Dunnett’s post-hoc test (results are presented in the Supplemental Information). Next, we analyzed for trends in drug response, a linear model was fitted to the dataset with dose values transformed to log scale (duration ~ log(dose)). Finally, to analyze drug interactions between hiPSC-aCMs and -vCMs, durations measured at each drug dose were normalized to percent change to baseline condition which were then analyzed using an unpaired t-test to compare the percentage change in duration of hiPSC-aCMs vs. -vCMs at each dose.

All data were presented as mean ± SEM (standard error of the mean) unless noted otherwise. Significance level for all statistical analysis was set at p < 0.05 with the following notation: *p < 0.05, **p < 0.01, ***p < 0.001. Statistical analysis and data visualization of optical mapping and NanoString data were conducted using R version 3.6.1. Statistical analysis of patch clamp recordings was performed using JMP 14.
